# A Motif Unique to the Human Dead-Box Protein DDX3 Is Important for Nucleic Acid Binding, ATP Hydrolysis, RNA/DNA Unwinding and HIV-1 Replication

**DOI:** 10.1371/journal.pone.0019810

**Published:** 2011-05-12

**Authors:** Anna Garbelli, Sandra Beermann, Giulia Di Cicco, Ursula Dietrich, Giovanni Maga

**Affiliations:** 1 Institute of Molecular Genetics, National Research Council, Pavia, Italy; 2 Georg-Speyer-Haus Institute of Biomedical Research, Frankfurt, Germany; University of Minnesota, United States of America

## Abstract

DEAD-box proteins are enzymes endowed with nucleic acid-dependent ATPase, RNA translocase and unwinding activities. The human DEAD-box protein DDX3 has been shown to play important roles in tumor proliferation and viral infections. In particular, DDX3 has been identified as an essential cofactor for HIV-1 replication. Here we characterized a set of DDX3 mutants biochemically with respect to nucleic acid binding, ATPase and helicase activity. In particular, we addressed the functional role of a unique insertion between motifs I and Ia of DDX3 and provide evidence for its implication in nucleic acid binding and HIV-1 replication. We show that human DDX3 lacking this domain binds HIV-1 RNA with lower affinity. Furthermore, a specific peptide ligand for this insertion selected by phage display interferes with HIV-1 replication after transduction into HelaP4 cells. Besides broadening our understanding of the structure-function relationships of this important protein, our results identify a specific domain of DDX3 which may be suited as target for antiviral drugs designed to inhibit cellular cofactors for HIV-1 replication.

## Introduction

The DEAD-box proteins constitute a subfamily of the DExD/H-box RNA helicases within the helicase superfamily 2 [1]. They are named after the amino acid sequence of the Walker B motif Asp-Glu-Ala-Asp (D-E-A-D), which is conserved in all members of the subfamily. The DEAD-box protein family comprises proteins found in all eukaryotes and most prokaryotes. Their widespread conservation is related to their essential roles in almost all aspects of RNA metabolism, from transcription, splicing and decay, to translation [2]–[4]. DEAD-box proteins are endowed with nucleic acid-dependent ATPase activity and some also show ATP-dependent RNA unwinding. While they differ in the amino acid (aa) sequence in their extreme N- and C- terminal parts, they all contain a core helicase domain, which is composed of two covalently linked globular domains (subdomain 1, aa 168–405 and subdomain 2, aa 411–582 in DDX3) each consisting of five β-strands and five α-helices, connected by a flexible linker (aa 406–410 in DDX3). Based on sequence alignments, nine conserved motifs have been identified in the helicase core of DEAD-box proteins. Motifs I (Walker A), II (Walker B) III and Q of the subdomain 1, together with motifs V and VI of subdomain 2, are directly involved in ATP binding and hydrolysis. Motifs Ia and Ib of subdomain 1 together with motifs IV, -[QxxR]- and V of subdomain 2 are involved in RNA binding [1]. Based on the available crystal structures of several DEAD box helicases showing the spatial arrangements of both domains, also in complex with RNA and nucleotides, a model has been proposed for the coordinated interplay of both subdomains in nucleic acid binding and ATP hydrolysis [5]. In the absence of bound ATP and nucleic acid, the DEAD-box proteins adopt open conformations, whereby subdomains 1 and 2 are separated from each other. On the other hand, the structures of the Vasa protein from *D. melanogaster*, human Dbp5 and DDX19, showed a closed conformation, with both subdomains creating an RNA binding surface and contributing to the ATP binding site [5]–[7]. Binding of ATP has been proposed to facilitate the interaction of the α-helix 8 of motif I with a conserved Arg in motif V, creating the RNA-binding competent conformation [5].

The DEAD-box ATPase/RNA helicase DDX3 has recently been in the focus of intense research, owing to its implications in tumor proliferation and viral infections [8]–[12]. In particular, DDX3 has been shown as an essential cofactor for human immunodeficiency virus type 1 (HIV-1) replication [13]. Functional genomic screenings have recently identified about 300 human proteins important for the replicative cycle of HIV-1 [14]. Such previously unrecognized complexity of host-pathogen relationships, coupled to the seemingly unavoidable problem of drug resistance due to the high mutation rate of HIV-1, has renewed the long-standing interest toward cellular proteins as novel targets for the treatment of HIV-1 infection. The main argument in favor of targeting a host factor instead of a viral protein is the predicted low drug resistance level. On the other hand, interfering with a cellular pathway might lead to serious complications, including crucial cell function loss or oncogenic drift. Thus, it is of paramount importance to identify host cellular factors and to characterize the domains that, while essential for virus replication, are dispensable for the host cell metabolism. The protein DDX3 apparently satisfies all the requirements of a potential anti-HIV target, and we have recently shown that small molecule inhibitors of DDX3 ATPase activity can block HIV-1 replication [12].

In order to design even more selective inhibitors of DDX3, a detailed knowledge of its biochemical properties is essential. DDX3 has been shown to have both ATPase and RNA helicase activities and to possess a rather relaxed nucleotide substrate specificity, being able to accept as substrates both ribo- and deoxy-nucleoside triphosphates, as well as different nucleotide analogs [11], [15], [16]. The core helicase domain (aa 132–605) of human DDX3 is nearly identical to *X. laevis* An3 and *M. musculus* Pl10 proteins and has strong homology to the corresponding domain of the yeast Ded1 and Dbp1 proteins [17]. Recently, the core domain of human DDX3 has been crystallized in complex with AMP [18]. In the absence of bound RNA, the protein adopts an open conformation. However, by analogy with other DEAD-box protein structures, it has been proposed that also DDX3 would adopt a closed conformation once bound to ATP and RNA.

**Figure 1:**
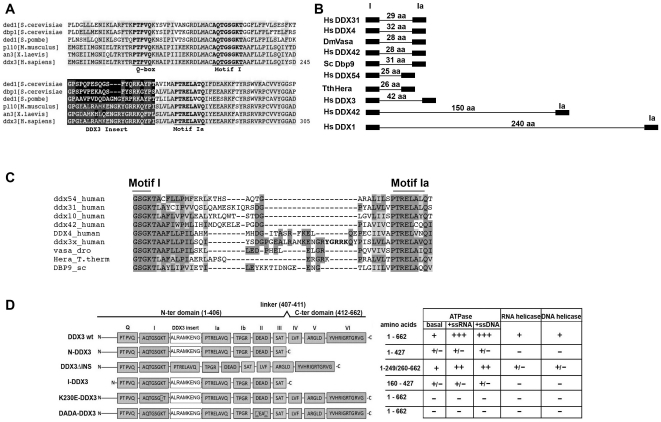
Human DDX3 has a unique insertion between motifs I and Ia. **A**. Multiple sequence alignment of the region between the Q-box and motif Ia of human DDX3 (accession n. NP001347) with *S. cerevisiae* Ded1p (accession n. P06634), *S. cerevisiae* Dbp1 (accession n. P2478), *S. pombe* Ded1 (accession n. O13370), *M. musculus* Pl10 (accession n. NP149068) and *X. laevis* An3 (accession n. NP001095245) proteins. Identical/equivalent amino acids are shaded. Residues of the conserved motifs are in bold. The DDX3 specific insertion is indicated with a black box. Multiple alignments were performed with the program ClustalW2 (www.ebi.ac.uk/Tools/clustalw2). **B**. Schematic representation of the length of the insertion sequences between motifs I and Ia of DDX3 and different DEAD-box proteins. The number of amino acids in each insertion is indicated. **C**. Multiple sequence alignment of the region between motif I and Ia of DDX3 and different DEAD-box proteins. Identical amino acids are shaded in dark grey, equivalent amino acids are shaded in light grey. Basic amino acids in the DDX3 insertion are in bold. Multiple alignments were performed with the program ClustalW2 (www.ebi.ac.uk/Tools/clustalw2). **D**. Schematic representation of the different mutants used in this study. The conserved motifs are named after their consensus sequence and represented as boxes. The name of the different mutants is on the left of the panel, while the corresponding amino acids are indicated on the right side, along with the corresponding biochemical properties revealed in this study. +, 100% (basal activity); ++, +++, stimulation of basal activity; +/−, reduced basal activity; −, no activity. Boxed residues in motifs I and II of the K230E and DADA mutants identify the single amino acid changes.

Human DDX3 has been reported to have a unique region between motifs I and Ia, which has been proposed to bind the 3′-end of the RNA substrate [18]. Such an insertion is also conserved in the closest homologs of DDX3 (Fig. 1 A). Sequence alignment analysis, revealed that the length of the amino acid sequence between motifs I and Ia is very similar among different DEAD-box proteins, being about 30 aa in representative human, insect, yeast and archeal proteins (Fig. 1 B). Among all eukaryotic DEAD-box proteins available in the databases, only three members appear to have longer insertions: DDX3 (42 aa), DDX42 (150 aa) and DDX1, this latter with the longest insertion of 240 aa. Sequence alignment of this region of DDX3 and other DEAD-box proteins, revealed a high degree of identity for the sequences immediately flanking the motifs I and Ia (Fig. 1 C), while the central part was more variable. Interestingly, the human proteins showed an invariant -[DG]-motif in common with DDX3, while proteins with more distant phylogenetic relationship (*Drosophila* Vasa, *T. thermophilus* Hera, *S. cerevisiae* Dbp9) lacked such a motif, but showed a -[ExG]- conserved box. The Vasa and Hera proteins also have in common with DDX3 an additional basic amino acid -[ExG**R**]- in this box. DDX3, however, was unique in having an additional positively charged extension -[YG**RRK**]-.

By using a series of different DDX3 mutants (Fig. 1 D), we show here that the central 10 residues of the DDX3 insertion (aa 250–259) are important for the ATPase activity, nucleic acid binding and unwinding. We also found that *in vitro* human DDX3 can unwind partially double stranded (ds) DNA substrates with a preference for 5′-single strand DNA overhangs, a property previously shown only for the *S.cerevisiae* Dbp9 member of the DEAD box proteins [19]. Finally, we show that the DDX3 unique region of the insertion between motifs I and Ia contributes to HIV-1 RNA binding and, furthermore, that a specific peptide ligand to this region reduces the ability of DDX3 to support HIV-1 replication in infected cells.

Taken together, our results uncovered novel unique features of human DDX3, which might be used to design novel selective anti-HIV agents.

## Results

### Construction, purification and ATPase activity determination of human DDX3 mutants

To study the role of the DDX3 specific insertion between motifs I and Ia, a deletion mutant, DDX3ΔINS, lacking aa 250–259 (Fig. 1 D and Materials and Methods) was constructed, starting from the cDNA for full length human DDX3. We also produced the N-DDX3 mutant lacking the C-terminus (Δ428–662) and consisting of the entire subdomain 1 plus the linker domain and the double deletion mutant I-DDX3 (Δ1–159/Δ428–662), corresponding to the helicase core of subdomain 1. Isolated domain 1 from different DEAD-box helicases has been successfully crystallized, indicating that the two domains can properly fold individually. Moreover, a multiple construct approach applied to DDX3 has previously shown that constructs lacking the first 169 aa were perfectly expressed as soluble proteins [18]. In addition, the single point mutant K230E, bearing a substitution in motif I (Walker A); and the double mutant DADA (D346A/D349A), bearing a double mutation in motif II (Walker B) were also constructed. The residues mutated in these proteins are known to be essential for ATP binding and hydrolysis [6], [13], which allowed us to include these loss-of-function mutants as controls to rule out the presence of any possible contaminating ATPase activity, as well as ATP-independent DNA unwinding activities. We also cloned the full length human DEAD-box protein DDX1, bearing the largest known insertion between motifs I and Ia. The corresponding cDNAs were cloned into the pTRcHis expression vector and expressed in *E. coli* as N-terminal his-tagged fusion proteins. The recombinant proteins were soluble and purified through four subsequent chromatographic steps, as described in Materials and Methods, involving affinity chromatography (Ni-NTA), anion- (MonoQ) and cation- (MonoS) exchange chromatography and hydroxyapatite (HAP) column.

**Figure 2:**
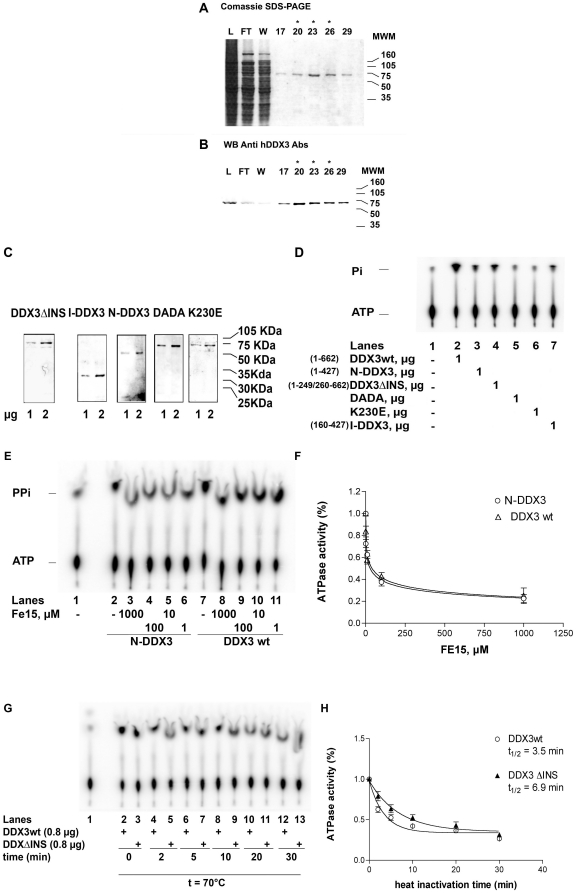
Purification of recombinant human DDX3. **A**. Coomassie staining of SDS-PAGE of the full length human DDX3 containing fractions eluted from the hydroxyhapatite column. L, loading; FT, flow-through; W, wash. Molecular weight is indicated on the right side. Fraction numbers are on top. Asterisks indicate the fractions used for the experiments. **B**. Western blot analysis of the fractions shown in panel A with anti-human DDX3 polyclonal antibodies. **C**. Coomassie staining of SDS-PAGE of the final purified preparation of all the recombinant DDX3 proteins. **D**. ATPase activity of all the recombinant DDX3 proteins. Reactions were performed as described in Material and Methods in the absence of nucleic acids. Unreacted substrate (ATP) was separated from the product (phosphate, Pi) by thin layer chromatography. The length (amino acids) of each protein is indicated in brackets. **E**. ATPase activity of N-DDX3 (lanes 2–6) or DDX3wt (lanes 7–11) in the absence (lanes 2 and 7) or in the presence of increasing amounts of the DDX3 inhibitor FE15. Lane 1, control without enzyme. **F**. Dose-response curves for the inhibition by FE15 of the ATPase activity of DDX3wt (circles) or N-DDX3 (triangles). Values are means of three independent determinations. Error bars are ±S.D. **G**. Representative time course experiment of the ATPase activity of DDX3wt (even lanes) or DDXΔINS (odd lanes) at 70°C. Lane 1, control without enzyme. **H**. Activity decay curves for the ATP hydrolysis by DDX3wt (circles) or DDX3ΔINS (triangles). Curves were fitted to a simple exponential decay model. Values are means of three independent determinations. Error bars are ±S.D.

As an example, Fig. 2 A shows the eluted peak fractions of DDX3wt from the last column (HAP). The protein in the fractions was recognized by specific anti-human DDX3 polyclonal Abs (Fig. 2 B). Fractions 20–26 (indicated with asterisks) were pooled and used for the experiments. Fig. 2 C shows the SDS-PAGE of all the other DDX3 proteins after purification. Next, the intrinsic ability of all the purified proteins to hydrolyze ATP in the absence of nucleic acid was tested. As shown in Fig. 2 D, the mutants lacking fully functional Walker A (K230E, lane 6) or Walker B (DADA, lane 5) motifs were catalytically inactive as expected. The DDX3wt and DDX3ΔINS mutant, on the other hand, displayed detectable ATPase activity (lanes 2 and 4). Surprisingly, the isolated subdomain 1 of DDX3, either full (N-DDX3, lane 3) or lacking the first 160 aa (I-DDX3, lane 7) also displayed low but detectable ATPase activity. To verify that ATP hydrolysis was catalyzed by the truncated mutants, and not due to a contaminant, we tested the sensitivity of the N-DDX3 mutant towards FE15, a specific inhibitor of DDX3 ATPase previously developed in our laboratory [12]. As shown in Fig. 2 E and F, ATP hydrolysis of both DDX3wt and the N-DDX3 mutant was inhibited by the compound FE15 with similar potency. To verify whether the introduced deletion could significantly alter the folding of the DDX3ΔINS mutant, thermal inactivation experiments were carried out. As shown in Fig. 2 G and H, DDX3ΔINS was about 2- fold more resistant to thermal inactivation than the wild type protein, as suggested by the time-dependent activity decay at 70°C. These results indicate that the specific 10 aa insertion of DDX3 likely plays a structural role, but is not essential for the intrinsic ATPase activity of the protein.

#### The DDX3 insertion between motifs I and Ia is required for stimulation of ATPase activity by DNA and RNA

**Figure 3:**
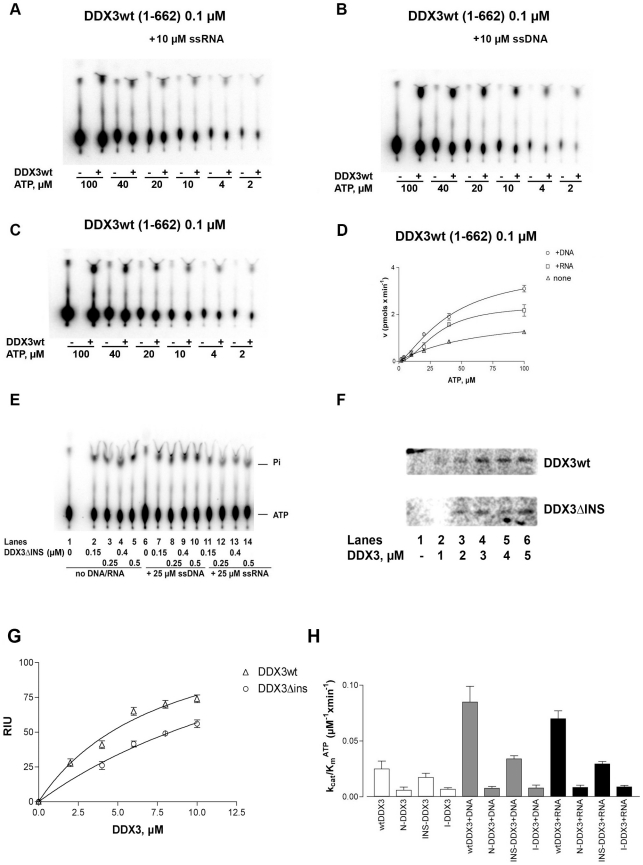
Characterization of the ATPase activity of recombinant human DDX3. Reactions were performed as described in Material and Methods. **A**. Product analysis of a representative experiment for the ATPase reaction catalyzed by 0.1 µM full length DDX3 in the presence of 10 µM ss RNA. **B**. As in panel A, but in the presence of 10 µM ss DNA. **C**. As in panel A, but in the absence of nucleic acids. **D**. Variation of the initial velocities of the reaction as a function of ATP concentrations, in the absence (triangles) or in the presence of 10 µM ss DNA (circles) or 10 µM ss RNA (squares). Data were fitted to Eq.(1) (see Materials and Methods). Values are the means of three independent determinations. Error bars are ±SD. **E**. Product analysis of the ATPase reaction catalyzed by increasing amounts of the DDX3ΔINS mutant in the absence (lanes 2–5) or in the presence of of 25 µM ss DNA (lanes 7–10) or ssRNA (lanes 12–15). Lanes 1, 6 and 11, control reactions in the absence of enzyme. **F**. [α-^33^P] ATP was UV crosslinked to increasing amounts of DDX3wt (upper panel) or ΔINS mutant (lower panel). Radioactive proteins were resolved on SDS-PAGE and revealed by phosphoImaging. **G**. Binding of DDX3 to ATP, as revealed by UV-crosslinking. Data were fitted to Eq.(5) (see Materials and Methods). Values are the means of three independent determinations. Error bars are ±SD. **H**. Comparison of the catalytic efficiencies (k_cat_/K_m_) for ATP hydrolysis of DDX3wt (aa 1–662), and the N-DDX3 (aa 1–427), DDX3ΔINS and I-DDX3 (aa 160–427) mutants, in the absence (white bars) or in the presence of ssDNA (grey bars) or ssRNA (black bars). Determination of the kinetic constants k_cat_ and K_m_ was performed as described in Materials and Methods. Values represent the means between two independent estimates of the k_cat_/K_m_ values from two sets of experiments. Error bars represent ±SD.

We have previously shown that the ATPase activity of wild type human DDX3 could be stimulated to a similar extent by a variety of different hetero- and homopolymeric nucleic acids (NA), irrespectively of their sequence or base composition, the main difference being the nature of the ribose sugar (RNA vs. DNA) [15]. Thus, we compared the ability of NA (both DNA and RNA) to stimulate the ATPase activity of the different DDX3 proteins. Addition of single strand (ss) RNA or DNA in the presence of DDX3wt clearly stimulated ATP hydrolysis (Fig. 3 A–C). The calculated kinetic parameters are reported in Table 1. DDX3 showed a higher apparent affinity for ATP (lower K_m_) as compared to that of most DEAD-box proteins [1]. The catalytic efficiency (k_cat_/K_m_) of DDX3wt was increased 2.8- and 3.2- fold by RNA and DNA, respectively (Table 1 and Fig. 3 H). The comparable stimulation of DDX3 ATPase by RNA and DNA was uncommon, since most DEAD-box proteins analyzed to date showed a preference for RNA *vs.* DNA as a cofactor of their ATPase activity [1]. The DDX3ΔINS mutant (lacking aa 250–259), showed a lower affinity for ATP than DDX3wt (Table 1), while its catalytic rate was similar. In order to assess whether the K_m_ value determined from the variation of ATP hydrolysis rate as a function of substrate concentration, truly reflected a difference in ATP binding, we measured the apparent equilibrium dissociation constant (K_D_) of ATP for DDX3wt and DDX3ΔINS, in the absence of catalysis, by directly UV-crosslinking [α^33^P]-ATP to the enzyme active site (Fig. 3 F). As shown in Fig. 3 G and Table 1, the K_D_ value for ATP was 2.8- fold higher for the DDX3ΔINS mutant than for DDX3wt, confirming a lower binding affinity of ATP for this mutant. The stimulation of the basal ATPase activity (k_cat_/K_m_) of this mutant by both RNA and DNA was lower than with the wt enzyme (Table 1 and Fig. 3 H), being less than 2- fold. These data suggest a possible role of the deleted domain in mediating the interaction with NA and with ATP, likely keeping the Walker A and B motifs correctly aligned for ATP binding. As shown in Fig. 3 E, increasing the DDX3ΔINS concentration from 0.15 µM to 0.5 µM did not result in better stimulation by either DNA or RNA. The N- and I-DDX3 mutants (lacking the entire subdomain 2), on the other hand, showed no stimulation of the ATPase activity in the presence of both NA (Table 1 and Fig. 3 F). As summarized in Table 1, the N- and I-DDX3 mutants also showed 3- to 9- fold reduced catalytic efficiency for ATP hydrolysis with respect to DDX3wt in the absence of NA. These data indicate that the isolated DDX3 subdomain 1 still retains a very low basal ATPase activity, while the presence of both subdomains is essential for full catalytic efficiency and stimulation by NA.

**Table 1:** ATPase reaction efficiency of DDX3wt and mutants and DDX1 in the absence or presence of nucleic acids.

Enzyme	K_D_^ATP^(µM)^a^	K_m_^ATP^(µM)^a^	*k*_cat_(min^−1^)^a^	*k*_cat_/K_m_(µM^−1^ min^−1^)
DDX3wt	7.5±2	62±8	1.6±0.2	0.025
DDX3wt+RNA^b^	n.d.	45±7	3.2±0.1	0.071
DDX3wt+DNA^b^	n.d.	47±7	3.8±0.2	0.08
N-DDX3	n.d.	101±15	0.8±0.3	0.008
N-DDX3+RNA	n.d.	85±15	0.98±0.03	0.011
N-DDX3+DNA	n.d.	120±20	1.1±0.5	0.009
I-DDX3	n.d.	110±20	1.1±0.2	0.01
I-DDX3+RNA^b^	n.d.	110±10	1.6±0.5	0.014
I-DDX3+DNA^b^	n.d.	100±9	1.4±0.4	0.014
DDX3Δins	21±5	115±15	2.1±0.5	0.018
DDX3Δins+RNA^b^	n.d.	90±10	2.7±0.3	0.03
DDX3Δins+DNA^b^	n.d.	81±11	2.7±0.3	0.033
DDX1	n.d.	120±20	1.9±0.3	0.016
DDX1+RNA	n.d.	90±10	3.8±0.5	0.042
DDX1+DNA	n.d.	95±14	2.1±0.3	0.022

a The kinetic parameters K_D_, K_m_, *k*_cat_, were determined by Eq.(1) and Eq. (5), as described in Materials and Methods. Values are the means of three independent replicates ± SD. K_m_ = K_D_+k_cat_/k_on_.

b RNA was oligo(rU)_20_; DNA was oligo(dT)_20_.

We also compared the ability of nucleic acids to stimulate the ATPase activity of the human DDX1 protein, that bears a long insertion (250 aa) between motifs I and Ia, but shows no similarity within this region with the equivalent region of DDX3. As shown in Table 1, the catalytic efficiency of DDX1 was stimulated 2.6- fold by RNA, but only 1.3- fold by DNA, as typically observed for DEAD-box proteins. Thus, the high degree of stimulation by DNA observed with DDX3 was not simply due to a wider distance between motifs I and Ia with respect to the majority of DEAD-box proteins (Fig. 1 B), but rather to sequence/structure-specific determinants.

#### The DDX3 insertion between motifs I and Ia is important for DNA binding

**Figure 4:**
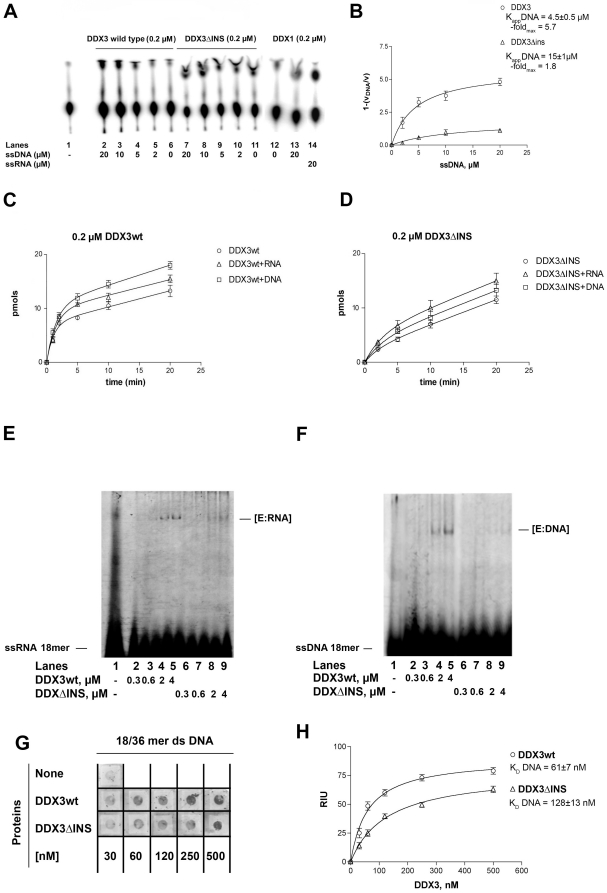
The specific insertion of human DDX3 is important for nucleic-acid stimulation of ATPase activity. Reactions were performed as described in Material and Methods. **A**. Product analysis for the ATPase reaction catalyzed by the DDX3wt (lanes 2–6) and the DDX3ΔINS (lanes 7–11) mutant proteins in the absence (lanes 6, 11) or in the presence of increasing concentrations of ssDNA. Lane 1, control reaction without enzyme. Reactions with the full length DDX1 protein (lanes 12–14) in the absence (lane 12) or in the presence of a fixed amount of DNA (lane 13) or RNA (lane 14), were included for comparison. **B**. Variation of the increase in the ATPase reaction rate (Δv) as a function of the ssDNA concentration in the presence of DDX3wt (circles) or DDX3ΔINS (triangles). The Δv values were derived as described in Materials and Methods. Data were fitted to Eq.(2) (see Materials and Methods). Values are the means of three independent determinations. Error bars are ±SD. **C**. Progress curves for the product formation during the ATPase reaction catalyzed by 0.2 µM of DDX3wt as a function of time, in the absence (circles) or in the presence of 10 µM ssRNA (triangles) or 10 µM ssDNA (squares). Data were fitted to Eq. (3) (see Materials and Methods). Values are the means of three independent determinations. Error bars are ±SD. **D**. As in panel C, but in the presence of 0.2 µM of the DDX3ΔINS mutant. **E**. Increasing amounts of DDX3wt (second row) or DDX3ΔINS mutant (third row), were incubated with a fixed concentration of (6-FAM)-5′-labelled ssDNA oligonucleotide. Nitrocellulose filter bound protein-DNA complexes were revealed by laser scanning. First row, control in the absence of proteins. **F**. Binding of DDX3 to ssDNA, as revealed by filter-binding assays. Data were fitted to Eq.(5) (see Materials and Methods). Values are the means of three independent determinations. Error bars are ±SD. **G**. Increasing amounts of DDX3wt (lanes 2–5) or DDX3ΔINS (lanes 6–9) were incubated in the presence of a (6-FAM)-5′-labelled ss RNA oligonucleotide. Enzyme-RNA ([E:RNA]) complexes were resolved by non denaturing PAGE and visualized by laser scanning. Lane 1, oligonucleotide alone.

In order to better characterize the ability of DNA to stimulate the ATPase activity of DDX3, the ATP hydrolysis rate was measured in the presence of increasing ssDNA concentrations. A typical experiment is shown in Fig. 4 A. Dose-response curves were generated by measuring the variation of the reaction velocity as a function of increasing ssDNA concentrations, in the presence of a fixed amount of ATP (Fig. 4 B). For each data point, the corresponding relative -fold increase values (1−v_DNA_/v) were plotted as a function of the nucleic acid concentrations and fitted to a hyperbolic relationship (see Materials and Methods). As shown in Fig. 4 B, the DDX3ΔINS mutant protein showed a significant reduction (3.2- fold) in the maximal stimulation of the ATPase activity by ssDNA, with respect to DDX3wt.

Next, the ATPase reaction of DDX3wt and the DDX3ΔINS mutant proteins was monitored at different time points in the absence or presence of both nucleic acids (NA) ssRNA and ssDNA. The corresponding progress curves were fitted to a mixed exponential equation (as detailed in Materials and Methods) as a function of time. This analysis allowed to estimate three parameters: the burst amplitude (A_burst_), which is proportional to the amount (expressed as a concentration) of the enzyme-substrates E:(NA):ATP complex formed at the beginning of the reaction; the burst rate (*k*_burst_) which represents the global rate of conversion of the E:(NA):ATP complex into the E:ADP complex and the steady-state rate (*k*_ss_), which is the limiting rate of the reaction at equilibrium (likely Pi release and NA dissociation). Thus, the effects of nucleic acids addition on the early steps of the reaction can be estimated by the variations of A_burst_ and *k*_burst_ values. Progress curves for DDX3wt and DDX3ΔINS are shown in Fig. 4 C and D. The calculated values are summarized in Table 2. Addition of RNA or DNA caused a 3- to 3.6- fold increase in A_burst_ values for DDX3wt. On the contrary, both RNA and DNA caused a smaller increase in the burst amplitude of the DDX3ΔINS mutant (1.8- to 2- fold increase). The *k*_burst_ of DDX3wt was also increased by nucleic acid addition (1.7- to 2.3-fold increase), while no effect on the *k*_burst_ for DDX3ΔINS was observed in the presence of DNA or RNA. The N- and I-DDX3 mutants showed reduced A_burst_ and *k*_burst_ values for ATP hydrolysis, with respect to wild type DDX3 in the absence of nucleic acids, indicating that the subdomain 1 alone was severely impaired both in binding and in hydrolysis of ATP. This is consistent with the important roles that residues in motifs V and VI are known to play in the formation of the ATP binding site. Neither DNA nor RNA had any effect on both A_burst_ and *k*_burst_ for the N- or I-DDX3 mutants, according to their loss of stimulation by NA.

**Table 2:** Kinetics of the ATP-dependent reaction of DDX3wt and mutants with nucleic acids.

Enzyme	A_burst_(µM)^a^	*k*_burst_(min^−1^)^a^	*k*_ss_(min^−1^)^a^
DDX3wt	0.25±0.05	4.1±0.7	0.3±0.08
DDX3wt+RNA^b^	0.75±0.1	7±0.8	0.4±0.09
DDX3wt+DNA^b^	0.9±0.1	9.5±0.8	0.4±0.03
N-DDX3	0.12±0.02	1±0.5	0.4±0.05
N-DDX3+RNA	0.13±0.01	0.5±0.5	0.35±0.06
N-DDX3+DNA	0.12±0.01	0.5±0.5	0.33±0.05
I-DDX3	0.12±0.02	0.3±0.4	0.3±0.05
I-DDX3+RNA^b^	0.11±0.03	0.4±0.5	0.4±0.04
I-DDX3+DNA^b^	0.15±0.02	0.4±0.6	0.3±0.05
DDX3Δins	0.12±0.05	0.4±0.1	0.2±0.03
DDX3Δins+RNA^b^	0.22±0.05	0.5±0.1	0.09±0.01
DDX3Δins+DNA^b^	0.24±0.05	0.5±0.1	0.12±0.01

a The A_burst_, *k*_burst_ and *k*_ss_ parameters were determined by Eq.(3) as described in Materials and Methods. Values are the means of three independent replicates ± SD.

b RNA was oligo(rU)_20_; DNA was oligo(dT)_20_.

Collectively, these results indicate that deletion of the specific DDX3 insert between motifs I and Ia reduced the formation of the enzyme-NA complex. In contrast, lack of the C-terminal subdomain 2 completely abrogated NA interaction.

From the curves shown in Fig. 4 B, the apparent affinity (K_app_) value for ssDNA was 3.3- fold higher for DDXΔINS compared to DDX3wt, suggestive of a reduced affinity for the nucleic acid. This K_app_ value, however, has been derived as a function of the ATP hydrolysis rate, thus being related to the concentration of ternary enzyme-DNA-ATP complex that has reacted with ATP and not directly to the enzyme-DNA binary complex formed. To further verify that the DDX3ΔINS had a reduced binding affinity for nucleic acids also in the absence of catalysis, an electrophoretic mobility shift assay was performed, in the presence of various amounts of DDX3wt or DDX3ΔINS and a fixed dose of a 5′-labelled ssRNA or ssDNA oligonucleotides. As shown in Fig. 4 E and F, the DDX3ΔINS was less efficient in binding RNA and DNA, respectively, with respect to DDX3wt, as judged by the lower amount of enzyme-NA complex formed (compare lanes 2–5 with lanes 6–9 in both panels E and F). Since DDX3 is also a helicase (see also below), dsRNA or DNA is also a physiological substrate. Thus, nitrocellulose filter-binding assays (Fig. 4 G) were performed to measure the affinity of DDX3wt and DDX3ΔINS to dsDNA, in the absence of ATP hydrolysis. As shown in Fig. 4 H, the DDX3ΔINS mutant showed a 2.1- fold reduced affinity for dsDNA, in agreement with the proposed role of the specific DDX3 insert in NA interaction.

### The human DDX3 specific insert is important for the RNA helicase activity

**Figure 5:**
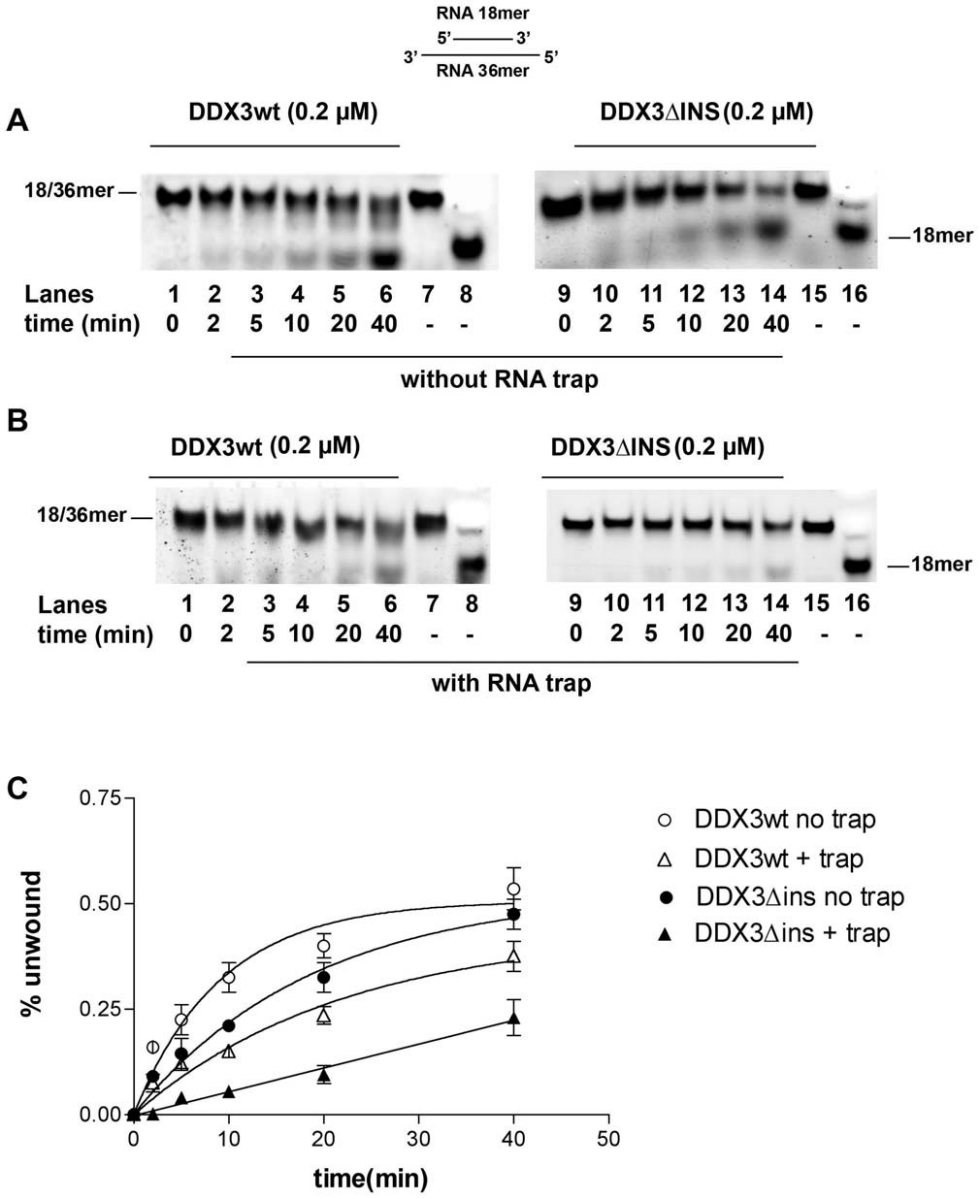
The specific insertion of human DDX3 is important for RNA unwinding. Reactions were performed as described in Materials and Methods. **A**. Representative RNA unwinding assay for DDX3wt, or the DDX3ΔINS mutant. Lanes 8, 16, boiled controls. **B**. As in A, but with the addition of a 50- fold molar excess of a r(A)_40_mer RNA trap ss oligonucleotide. **C**. Quantitative analysis of the RNA unwinding reaction catalyzed by DDX3wt and the DDX3ΔINS mutant. Values are the means of three independent determinations. Error bars are ±SD.

Human DDX3 was shown to possess an ATP-dependent RNA helicase activity [12], [13], [15]. The ability of the different DDX3 mutants to unwind a double-strand (ds) RNA heteropolymeric template was therefore compared. As shown in Fig. 5 A, DDX3wt (lanes 1–6) showed more efficient RNA helicase activity than the DDX3ΔINS mutant (lanes 9–14). The previous experiments (Fig. 4 and Table 2), suggested that this mutant had an impaired RNA binding. To test whether this reduced affinity for RNA was the cause of the reduced helicase activity, the same experiments were performed under single-turnover conditions, i.e. in the presence of a 50-fold molar excess of unlabelled ssRNA as a trap. Under these conditions, as soon as the enzyme dissociates from the duplex substrate, it should be sequestered by the competitor RNA preventing rebinding to the labelled substrate. As shown in Fig. 5 B, while DDX3wt (lanes 1–6) was still able to unwind the RNA duplex in the presence of the trap, the activity of the DDX3ΔINS mutant (lanes 9–14) was almost completely inhibited in the presence of the competitor RNA. This suggests that the complex between this mutant and the RNA duplex had a significantly reduced half-life with respect to the wt enzyme, becoming sequestered by the competitor ssRNA before it could initiate the unwinding reaction. The corresponding progress curves are shown in Fig. 5 C and the calculated apparent rates of unwinding (*k_uw_*) are reported in Table 3. Addition of the trap reduced the unwinding rate of DDX3wt by about 2- fold, suggesting that under distributive conditions (i.e. without trap) DDX3wt required two binding events in order to fully displace the 18mer strand from the complementary 36mer. In the absence of the trap, the DDX3ΔINS mutant showed a 2- fold reduced unwinding rate with respect to DDX3wt, while addition of the trap caused a 10-fold reduction in the unwinding rate (Table 3). Thus, the DDX3ΔINS mutant was significantly impaired in its RNA unwinding activity. As expected, the isolated subdomain 1 (N- or I-DDX3) did not show helicase activity (data not shown). These results clearly indicate that both subdomains are required for helicase activity and that the DDX3 insertion between motifs I and Ia is important for interaction with RNA.

**Table 3:** Kinetics of RNA unwinding by DDX3wt and the DDX3ΔINS mutant.

Enzyme	*k*_uw_(min^−1^)^a^
**DDX3wt**	0.11±0.02
**DDX3wt+trap**	0.05±0.01
**DDX3ΔINS**	0.05±0.01
**DDX3ΔINS+trap**	0.005±0.001

a The *k_uw_* values were was estimated by Eq.(4) as described in Material and Methods. Values are the means of three independent replicates ± SD.

#### Human DDX3 can unwind partially double-stranded DNA with 5′-single strand overhangs

**Figure 6:**
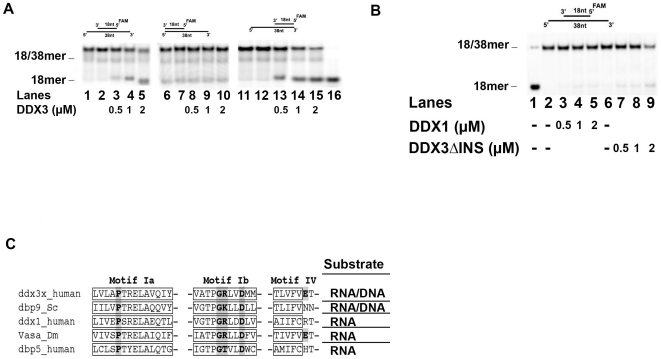
Human DDX3 can act as a DNA helicase. Experiments were performed as described in Materials and Methods. The 18-mer oligonucleotide was fluorescently labelled at its 5′-end in all the experiments. The position of the unreacted substrate (18/38-mer) and of the released strand 18-mer are indicated on the left side of each panel. **A**. Strand-displacement reactions catalyzed by increasing amounts of DDX3wt in the presence of the different partially dsDNA substrates: 18/[5′-3′]-38 mer (lanes 1–5); 18/[3′]-38 mer (lanes 6–10); 18/[5′]-38 mer (lanes 11–15). Lanes 1, 6, 11: control reactions incubated in the absence of enzyme. Lanes 2, 7, 12: reaction mix with no enzyme loaded on gel without incubation. Lane 16: boiled substrate. **B**. Strand displacement activity of increasing amounts of the DDX1 (lanes 3–5) or DDX3ΔINS (lanes 7–9) proteins on the 18/[5′-3′]-38 mer substrate. Lanes 2, 6, control reactions incubated in the absence of enzyme; lane 1, boiled substrate. **C**. Sequence alignment of human DDX3, *S. cerevisiae* Dpb9, human DDX1, *D. melanogaster* Vasa and human Dpb5, showing the structural motifs Ia, Ib and IV. Residues known to mediate specific 2′-OH ribose contacts in the Vasa crystal structure are highlighted. The corresponding helicase substrates for each enzyme are indicated on the right side of the panel.

The ability of the human DDX3 ATPase to be significantly stimulated by DNA prompted us to investigate whether the enzyme was able to unwind ds DNA substrates. To this aim, we employed three pairs of partially complementary DNA oligonucleotides, to generate partially dsDNA substrates with either 5′- or a 3′- protruding ss ends, or both (Fig. 6 A). The shorter oligo was 5′-labelled with a fluorescent group (FAM). Helicase reactions were carried out in the presence of ATP and the products resolved on non-denaturing PAGE. Human DDX3wt was able to unwind a DNA duplex carrying both 5′- and 3′-protruding ssDNA ends (Fig. 6 A, lanes 1–5) or with a single 5′-protruding end (lanes 11–15), but not the substrate with a 3′-ss DNA region (lanes 6–10). As expected, mutants lacking ATPase activity (K230E and DADA) or DNA binding (N- and I-DDX3) were found to be inactive also as DNA helicases (data not shown). The mutant DDX3ΔINS showed DNA helicase activity, but much lower than DDX3wt (compare Fig. 6 A, lanes 3–5, with Fig. 6 B, lanes 7–9), consistent with its reduced affinity for DNA (shown in Fig. 4). Human DDX1, similarly to most DEAD-box helicases, did not display DNA unwinding capability (Fig. 6 B, lanes 3–5). Taken together, these data suggest that lack of the specific DDX3 insert (aa 250–259) causes a reduction in DNA unwinding, while the mere presence of a long insertion between motifs I and Ia (as is the case for DDX1) did not suffice to confer DNA helicase activity.

#### The DDX3 specific insertion is important for the function of DDX3 as a cofactor of HIV-1 replication

**Figure 7:**
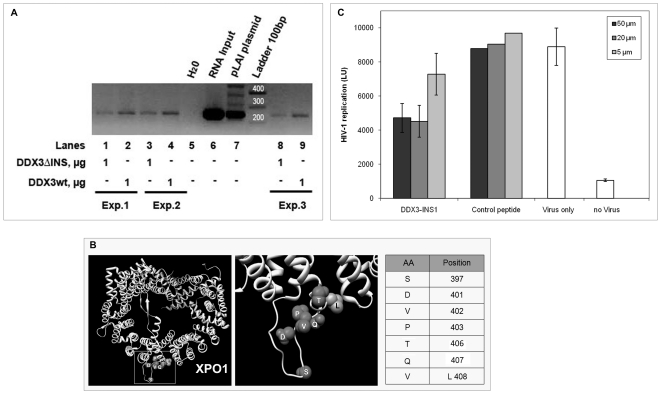
The DDX3 insert is functionally implicated in the HIV-1 cofactor activity of DDX3. **A**. HIV genomic RNA pull down experiments with purified DDX3wt and DDX3ΔINS proteins. Bound HIV RNA was detected by RT-PCR with *gag*-specific primers in three independent experiments (lanes 1 and 2; lanes 3 and 4; lanes 8 and 9). Lanes 6 and 7 correspond to RT-PCR fragments of input viral RNA and PCR of the HIV-1 pLai plasmid, respectively; lane 5 is the negative PCR control. **B**. Peptide DDX3-INS1 selected with the DDX3 insertion by phage display shows homology to XPO1. The crystal structure of XPO1 (DOI:10.2210/pdb3gb8/pdb) is shown and amino acids identical to the peptide sequence are represented as spheres. The corresponding positions are indicated on the right side of the panel, along with the conservative substitution V408L. **C**. Antiviral activity of the selected peptide DDX3-INS1 fused to a protein transduction domain. Peptides DDX3-INS1 and a control peptide were transduced into HelaP4 cells after infection with HIV-1Lai and virus in supernatants was quantified on Tzm-bl cells by luminometry after 44 hours. Bars represent the mean of 3 independent experiments and error bars indicate SD. For the control peptide, one representative experiment is shown.

A role for DDX3 in the export of HIV-1 unspliced RNA, in cooperation with the vial protein Rev and the cellular protein XPO1/CRM1 has been proposed [13]. In order to analyze the effect of the DDX3 specific insertion in view of this role of DDX3 as cellular cofactor for HIV-1 replication, we first performed HIV-1 RNA pulldown experiments, using viral genomic RNA prepared from HIV-1 virions with equal amounts of purified DDX3wt and DDX3ΔINS proteins. Bound viral RNA was quantified by RT-PCR of a fragment corresponding to the *gag* gene. Fig. 7 A summarizes the results of three independent experiments. Clearly, amplified *gag* fragments were reproducibly weaker for DDX3ΔINS than for DDX3wt suggesting a contribution of the DDX3 insertion in binding of HIV-1 genomic RNA.

To further analyze the role of the DDX3 insertion in the context of HIV-1 replication, we selected peptide ligands for the DDX3 insertion from phage displayed random peptide libraries. Interestingly, one of the selected peptides, DDX3-INS1, showed amino acid homologies to XPO1/CRM1, a natural binding partner of DDX3 that is involved in the export of unspliced HIV-1 RNA in conjunction with the viral Rev protein (Fig. 7 B). We therefore analyzed this peptide further, with respect to functional interference with HIV-1 replication. After fusion to a protein transduction domain, different amounts of the DDX3-INS1 peptide or a control peptide were transduced into HelaP4 cells 24 hours after infection with HIV-1Lai. HIV-1 infection was quantified 48–72 hours after infection by titering released HIV-1 in culture supernatants on Tzm-bl cells based on measuring the luciferase activity in the cell lysates. As shown in Fig. 7C, the DDX3-INS1 peptide reduces HIV-1 replication about 50% at 20 µM in contrast to the control peptide. The DDX3-INS1 peptide was not toxic for the cells in the concentration range used (data not shown). Thus, binding of the peptide ligand to the DDX3 insertion, interferes with HIV-1 replication further providing evidence of an important functional role of the DDX3 insertion not only for enzymatic functions, but also for HIV-1 replication.

## Discussion

Human DDX3 was co-crystallized with AMP in the absence of nucleic acid and thus the available structure represents the “open” conformation of the protein, as opposed to the “closed” conformation observed for other DEAD box proteins in complex with RNA, which involves repositioning of subdomains 1 and 2 relative to each other to accommodate both RNA and ATP substrates [18]. This fact, together with the presence of AMP and not ATP in the binding site of DDX3, did not allow to establish a role of domains V and VI in mediating the interactions with the β- and γ- phosphates of the nucleotide as seen in the RNA-bound structures of the VASA protein of *D. melanogaster* [6] or the human Dbp5 [7]. Our results showed that the isolated subdomain 1 alone (mutant N-DDX3 aa 1–427) of human DDX3 possesses a low but detectable intrinsic (i.e., nucleic acid-independent) ATPase activity. This finding is apparently in contrast with the reported lack of ATPase activity of the entire DDX3 helicase core (aa 168–582) used for crystallization [18]. It is possible that the isolated subdomain 1 might fold more easily into a catalytically active globular domain.

On the basis of the structures of Vasa and Dbp5 [6], [7], it has been proposed that a conserved aromatic residue (F or Y) of motif VI (-GR**F**/**Y**G-) stacks on the purine ring of adenosine, thus contributing to its binding. Sequence alignment showed that this residue is not conserved in DDX3, where it is replaced by a valine (-GR**V**G-). On the other hand, the conserved aromatic residue of the Q-motif, which stacks also on top of the purine ring, is conserved in DDX3 (Y201). A metal ion is coordinated to the β- and γ- phosphates of ATP, through essential water molecules which are contacted by the invariant aspartic acid of Walker B and a glycine residue of motif VI. Both these residues are also conserved in DDX3 and, based on the overall high homology between the conserved motifs of Vasa and DDX3, they have been proposed to play a similar role as seen in Vasa [18]. Other specific contacts of subdomain 2 include the ribose (motif V) and the triphosphate moiety (motif VI) of ATP. However, the structures of Vasa and Dbp5 clearly indicated that the majority of the interactions, including those occurring with the water molecules, are mediated by residues of motifs Q, I, II and III, all confined in the N-terminal subdomain 1. Since a structure for DDX3 in its closed conformation is lacking, it is possible that the DDX3 subdomain 1 alone might adopt a folding, which enables stable nucleotide binding and hydrolysis, even though at a very low rate when compared to the wild type protein, as indicated by our data. Our results clearly indicate an essential contribution of subdomain 2 in increasing the intrinsic ATPase activity as well as in mediating nucleic acid binding and stimulation of ATP hydrolysis, as seen in all known DEAD-box proteins.

Both the ATPase and nucleic acid binding activities of the DEAD-box proteins are functionally connected to their helicase activity. In this respect, DEAD-box proteins are considered to behave more as ATP-dependent RNA binding proteins, than classical helicases, since ATP binding/hydrolysis does not generate energy for translocation, but rather drives the conformational changes required to destabilize the double helix [20]–[23]. Based on the DDX3 crystal structure, it has been proposed that the specific insertion (aa 250–259) seen in DDX3 between motifs I and Ia could contribute to nucleic acid binding, together with the adjacent unique basic motif [-RYGRRK-] [18]. We have experimentally proven this hypothesis by showing that the DDX3 mutant lacking this particular insertion has a seriously compromised RNA and DNA ATPase stimulation and unwinding activity. In addition, the deleted insertion likely plays some structural role in defining the architecture of the ATP binding site, since the DDX3ΔINS mutant displayed reduced ATP binding with respect to DDX3 full length.

We found that human DDX3, contrary to most DEAD-box helicases, can unwind partially dsDNA substrates with a preference for DNA substrates containing a 5′-single stranded overhang. DNA unwinding by a DEAD-box protein has been shown so far only for the Dpb9 protein of *S. cerevisiae* [19]. The strong preference for RNA by DEAD-box proteins has been ascribed to four specific ribose 2′-OH contacts, mediated by five amino acids in motifs Ia, Ib and IV, as seen in the Vasa crystal structure. Sequence alignment of human DDX3, *S. cerevisiae* Dpb9, human DDX1, *Drosophila* Vasa and human Dbp5 (Fig. 6 C), showed that the residues in motif Ia and Ib are conserved in all proteins, irrespectively of their RNA vs. DNA preference, while the glutamic acid residue of motif IV is present only in DDX3 and Vasa. Thus, it seems that the presence of these residues did not suffice to confer an absolute preference for RNA *vs* DNA to DEAD box proteins. A model of DDX3 in the closed conformation bound to RNA, based on the Vasa-RNA complex structure, suggested that the specific DDX3 insertion between motifs I and Ia, contributes to generate a positively charged cavity in the close proximity of the 3′-end of the bound nucleic acid [18]. The 5′-strand loading preference observed for DDX3 DNA unwinding implies that the annealed strand is displaced starting from its 3′-end (Fig. 6 A). It is then tempting to speculate that the unique DDX3 insertion might partially contribute to the 5′-end loading preference of DDX3. It is interesting to note that the human protein DDX1, which bears a very large insertion (250 aa) between motifs I and Ia, has a clear preference for RNA as an activator for its ATPase and does not show DNA helicase activity (Fig. 4 A and 6 B).

Furthermore, we could experimentally prove that the DDX3 insertion functionally contributes to the cofactor activity of DDX3 with respect to HIV-1 replication (Fig. 7). First, lack of the insertion in DDX3ΔINS reproducibly resulted in reduced pull down of HIV-1 genomic RNA compared to DDX3wt as detected by RT-PCR. Second, a specific peptide ligand, selected from phage display libraries with a DDX3-derived peptide containing the insertion, was shown to interfere with replication of HIV-1 Lai in HelaP4 cells. Interestingly, the selected peptide showed extensive homology to XPO1/CRM1, a natural binding partner of DDX3 involved in nuclear export of unspliced HIV RNAs in conjunction with Rev. It is possible that, by binding to the insertion between domains I and Ia of DDX3, the peptide prevented the correct assembly of the DDX3/CRM1/Rev/HIV-RNA complex, required for nuclear export. Additional experiments with optimized peptides have to further elucidate if, indeed, interference with viral replication occurs at the level of RNA export from the nucleus. In fact, a recent study revealed a role of DDX3 also in the translation of HIV-1 proteins [24].

In summary, we have provided evidence for a role of the unique motif located in the DDX3 N-terminal subdomain 1 in ATPase activity, nucleic acid binding and HIV-1 cofactor activity of DDX3. Given the interest of DDX3 as a possible target for antiviral chemotherapy, the design of small molecules specifically interacting with this DDX3 unique motif might be considered.

## Materials and Methods

### Nucleic Acids

The PCR primers, the oligo(dT)_20_ and oligo(rU)_20_ used for the stimulation of the ATPase activity and the DNA oligonucleotides used for the helicase assays were synthesized by MWG-Biotech (Florence, Italy) and PAGE purified.RNA oligonucleotide sequences were:r18mer_FAM: 5′CCCAAGAACCCAAGGAAC-3′ *r36mer:5′ACCAGCUUUGUUCCUUGGGUUCUUGGGAGCAGCAGG-3′*. The r36 mer oligonucleotide bears a sequence complementary to the 18 mer (in italic). The r18 mer was labelled at its 5′ end with a fluorescent group (6-FAM).

DNA oligonucleotide sequences were:d18mer_FAM:5′CTCAACTCAACCCTTCAT-3′d38mer_central:5′AATATTGAGG*ATGAAGGGTTGAGTTGAG*TGGAGATAGT-3′d38mer_5′: 5′*ATGAAGGGTTGAGTTGAG*TGGAGATAGTGGAGGGTAGT-3′d38mer_3′:5′GGAGGGTAGGAATATTGAGG*ATGAAGGGTTGAGTTGAG*-3′

The 38 mer oligonucleotides bear a sequence complementary to the 18 mer (in italic) at different positions. The 18 mer was labelled at its 5′ end with a fluorescent group (6-FAM).

### Chemicals

[γ-^32^P] ATP (3000Ci/mmol) was from GE Healthcare. [α-^33^P] ATP (3000Ci/mmol) was from Hartmann Analytic GmbH. Unlabelled ATP was from Sigma. All other reagents were of analytical grade and purchased from Merck or Fluka.

### Cloning of human DDX3

The human DDX3 coding sequence was cloned from PBMC cells as described [15].

### Construction of DDX3 mutants

Starting from the full-length cDNA construct, the N-DDX3 (aa 1–427), DDX3ΔINS (aa 1–249/260–662) and I-DDX3 (aa 160–427) mutants were constructed by PCR. The N-DDX3 mutant (aa 1–427) was obtained using the forward primer 5′-ATACGGATCCTGCATGAGTCATGTGGCAGTG-3′ and reverse primer 5′-CCCAAGCTTTTTGTCTGATTCTTCCACCCAAACTACTTTCTG-3′. The double deleted mutant I-DDX3 (aa 160–427) was obtained by PCR using the forward primer 5′-GGGGGATCCTTTGAGAAATACGATGACATTCCAGTTGAG-3′ and reverse primer 5′-CCCAAGCTTTTTGTCTGATTCTTCCACCCAAACTACTTTCTG-3′. The ΔINS mutant (Δ250–259), was obtained by overlap extension gene split PCR, where the fragment coding for aa 1–249 was amplified using the forward primer 5′-ATACGGATCCTGCATGAGTCATGTGGCAGTG-3′ and the reverse primer 5′-TGCTAATACCAAGGAGATTGGCAAGATGGGCAACAGAAATGC-3′, while the fragment coding for aa 260–662 was amplified with the forward primer 5′- CCAATCTCCTTGGTATTAGCA-3′ and the reverse primer 5′-ATCGAAGCTTGTTACCCCACCAGTCAACC-3′. The two resulting fragments had a short region of complementarity at their 3′- and 5′- ends, respectively and were used in a third PCR reaction with the forward primer 5′-ATACGGATCCTGCATGAGTCATGTGGCAGTG-3′ and the reverse primer 5′-ATCGAAGCTTGTTACCCCACCAGTCAACC-3′, to generate the desired cDNA for the ΔINS mutant. PCR conditions for all mutants were: 2′ at 95°C followed by 30 cycles of 40″ at 95°C, 1′ at 55°C, 1′30″ at 72°C and a final elongation of 10 min at 72°C. The K230E DDX3, with a substitution of the lysine at the position 230 with a glutamic acid, and the DADA DDX3 mutant, with a substitution of the two aspartic acids at positions 328 and 231 for alanines, were purchased from Bio-Fab Research (Rome). All cDNAs were sequenced for confirmation and cloned into the pTrcHisA vector, resulting in the desired protein fused to an N-terminal peptide of ∼3 kDa (comprising the 6×His tag) and seven additional amino acids (KQGCFGG) at the COOH-terminal part.

### Expression and purification of the recombinant proteins

All clones were expressed in *E.coli* competent cells BL-21 Select^96^ (Promega). Small-scale expression experiments were carried out to select the optimal conditions for solubility of the expressed proteins. Cells were grown at 37°C in Luria Broth (LB) to an OD_600_ of 0.4. Isopropyl β-D-thiogalactoside (IPTG) was added to a final concentration of 1 mM and growth continued for 5 hours at 37°C. Recombinant proteins were purified through four different chromatographic steps. Cells were lysed for 45′ on ice adding 2.5 volumes per gram of pellet of Buffer A (0.1 M NaPO_4_, 0.01 M TrisHCl pH 8, 0.01%, 5 mM NP-40 Imidazole) in the presence of lysozyme (1 mg/ml), protease inhibitors (1∶10000 dilution, Sigma) and 1 mM PMSF. The lysate was sonicated 5 times for 10″ and the suspension was centrifugated at 30000× g for 1 h15′ at 4°C. The supernatant was applied to an affinity FPLC-NiNTA-column, equilibrated with Buffer B (5 mM TrisHCl 2 pH 8, 250 mM NaCl, 5 mM Imidazole, 5% Glycerol). Proteins bound to the column were eluted with a linear gradient (0.005–0.5 M Imidazole) in Buffer B. The presence of the recombinant protein in the collected fractions was checked by staining (Coomassie Blue) and Western blotting with specific anti-DDX3 antibodies (A300–474A and A300–475A, Bethyl) that recognized an epitope at N-terminal domain between residues 1 and 50 and at the C-terminal domain between residues 550 and 600, respectively. For the I-DDX3 mutants we have used anti-His tag polyclonal antibodies (Cell Signaling TECHNOLOGY®). Positive fractions were collected and dialyzed against Buffer C (25 mM TrisHCl pH 7.5, 10 mM NaCl, 5% glycerol and 0.5 mM L-Dithiothreitol (DTT)) at 4°C. Dialyzed fractions were applied to an anion-exchange Mono Q column previously equilibrated with Buffer C and eluted with a linear gradient (0.01–1 M NaCl) in Buffer C containing 1 M NaCl. After SDS-PAGE and Western blot, positive fractions were pooled and dialyzed against Buffer D (50 mM TrisHCl pH 7.5, 10 mM KCl, 0.5 mM DTT, 5%Glycerol). Dialyzed fractions were applied to a cation-exchange chromatography (MonoS) column equilibrated with Buffer D and proteins bound were eluted with a linear gradient (0.01–0.5 M KCl) in Buffer D containing 500 mM of KCl. Positive fractions were collected and dialyzed against Buffer C. Dialyzed fractions were applied to a 1-ml hydroxyapatite column equilibrated in Buffer C. Proteins were eluted with a linear gradient (0–0.7 M KPO_4_, pH 7.5) in Buffer C containing 0.7 M KPO_4_ pH 7.5. Positive fractions were made 20% glycerol and stored at −80°C.

### ATPase activity assays

The ATPase activity was determined, as previously described [15], by directly monitoring [γ-^32^P] ATP hydrolysis by thin-layer chromatography (TLC). A final volume of 10 µl contained: 0.1 µM of the different DDX3 proteins, unless otherwise indicated in the Figures, 0.1 µM [γ-^32^P] ATP (3000 Ci/mmol) as tracer, plus 1 mM of cold ATP, unless otherwise stated, and nucleic acids as indicated. Samples were incubated for 5 minutes at 25°C and dotted onto TLC sheets of polyethylenimine cellulose. The products were separated by ascending chromatography with 0.5 M KH_2_PO_4_ (pH 3.4). The intensities of the radioactive bands corresponding to ATP and P_i_ were quantified by densitometric scanning with PhosphoImager. For the stimulation of the ATPase activity, the ssDNA oligonucleotide oligo(dT)_20_ or the ssRNA oligonucleotide oligo (rU)_20_ were used. Time-course experiments were performed under the conditions described above. Samples were taken at different time points and processed as described above.

### ATP binding assay

ATP binding assays of DDX3wt and DDX3ΔINS proteins were performed by UV cross-linking. 20 µl samples containing different amounts of purified proteins were resuspended in reaction buffer (35 mM Tris-acetate pH 8, 70 mM potassium acetate, 5 mM magnesium acetate, 19 mM ammonium acetate, 0.7 mM DTT) containing 7 µCi of [α-^33^P]ATP (110TBq/mmol) together with cold ATP up to a final concentration of 0.5 µM. Samples were subjected to UV irradiation (254 nm) for 15 minutes on ice. Samples were boiled 5 minutes at 95°C and separated by SDS-PAGE on a 12% polyacrylamide gel. The gel was exposed in a phosphorimager storage cassette for 16–18 hours and quantified by laser scanning densiometry (Thyphoon-TRIO, GE Healthcare).

### Filter binding assay

The affinity of DDX3wt and DDX3ΔINS proteins for dsDNA was assessed using a filter binding assay. Different amounts of proteins were incubated in reaction buffer (10 mM Hepes pH 7.2, 100 mM KCl, 3 mM MgCl_2_, 5% glycerol, 1 mM DTT, 50 µg/ml salmon sperm DNA). The 18/36 mer dsDNA (labelled at 5′-end of one strand with a 6-FAM fluorescent group) concentration was kept fixed at 50 nM. The binding reactions (25 µl) were incubated for 14 15 minutes at 24°C. Assays were performed using the protein-binding Protran BA-85 nitrocellulose membrane. The membrane was extensively washed in washing buffer (10 mM HEPES pH 7.2, 3 mM MgCl_2_, 5% glycerol, 1 mM DTT), and mounted on a 96-well dot-blotter (Bio-Rad). Before and after application of 25 µl of the binding reaction, the membrane was washed with 180 µl of wash buffer. Following the experiment, the membrane was dried and the quantity of bound proteins determined by laser scanning densiometry (Thyphoon-TRIO, GE Healthcare).

### Electrophoretic mobility shift assay (EMSA)

The affinity of DDX3wt and DDX3ΔINS proteins for ssDNA and ssRNA was assessed using the EMSA assays. Different amounts of proteins were transferred to a fresh pre-cooled Eppendorf tube and the reaction volume was increased to 15 µl with protein buffer (25 mM TrisHCl pH 7.5, 0.5 mM DTT, 20% glycerol. 100 mM NaCl). 4 µl of 5× EMSA binding buffer (10% glycerol, 5 mM MgCl_2_, 2.5 mM EDTA pH 8, 50 mM NaCl, 50 mM TrisHCl pH 7.5, 10 mM DTT) was added to the samples, which were incubated on ice for 20 minutes. After this time, 0.05 pmol of ssDNA or ssRNA (labelled at 5′-end with a 6-FAM fluorescent group) was added to the reactions and the samples kept on ice for 1 hour. The free- and protein bound-nucleic acids were separated on a non-denaturing polyacrilamide gel (6%), run at a constant 10 V/cm for 2 hours. Substrates and products were quantified by laser scanning densitometry (Thyphoon-TRIO, GE Healthcare).

### Helicase Assays

The helicase activity of DDX3wt and mutant forms was monitored by measuring the conversion of a double stranded (ds) DNA or RNA (labelled at the 5′-end of one strand with a 6-FAM fluorescent group) into single stranded (ss) nucleic acid. For the RNA helicase assay, a 18/36mer ds RNA oligonucleotide with both 3′ and 5′-protruding ss ends was used. For the DNA helicase assay, three different dsDNA substrates were used, all of them composed of the same 18mer oligonucleotide 5′-FAM labelled and three different 38 mer oligonucleotides with the sequence complementary to the 18 mer at different positions. Annealing of the 18 mer to each of the 38 mer generated the different DNA substrates shown in Fig. 5 A. A final concentration of 20 nM RNA or DNA substrate was used in the experiments, unless otherwise stated. The amounts of the different DDX3 proteins are indicated in the respective Figures.

Reactions were performed in 50 mM TrisHCl pH 7.5, 1 mM DTT, 0.2 mg/ml BSA, 5% glycerol and 1 mM ATP, 10 mM MgCl_2_ at 37°C degrees for 10′ and stopped by adding EDTA 50 mM pH 8. Products were separated on a native 7% PAGE containing 0.1% (w/v) SDS at 5 W in TBE buffer with 0.1% SDS at 4°C for 2 hours for RNA substrates, 4 hours for DNA substrates. Substrates and products were quantified by laser scanning densitometry (Thyphoon-TRIO, GE Healthcare). For trap-assays, the enzyme and the RNA substrate were incubated on ice for 4 min, in the absence of ATP. Reactions were started by addition of ATP and a 50-fold molar excess (1 µM) of r(A)_40_ trap RNA oligonucleotide, followed by incubation at 37°C for the indicated times.

### Kinetic analysis

ATPase reactions were performed as described, in the presence of increasing amounts of nucleic acids and variable ATP concentrations. Variations of the initial velocities of the reaction as a function of ATP concentrations in the absence or presence of nucleic acids were analyzed with the equation:
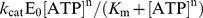
(1)where *k*_cat_ is the reaction rate, E_0_ is the input enzyme concentration, [ATP] is the variable substrate concentration, *K*_m_ is the apparent affinity for the substrate, *n* is the sigmoidicity factor, to take into account the apparent positive cooperativity previously observed for DDX3 in response to increasing ATP concentrations [12]. The relative -fold increase in the ATPase velocities 1−(v_DNA_/v) as a function of the nucleic acid concentrations was fitted to the hyperbolic relationship:
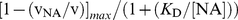
(2)where v_NA_ is the apparent velocity of the reaction in the presence of nucleic acid, v is the velocity in its absence, K_D_ is the apparent affinity for the nucleic acid and [NA] is the concentration of the nucleic acid substrate. The symbol K_D_ is used, instead of K_m_, solely to reflect the fact that the NA is not a reactant (i.e. it is not converted into products) but only a cofactor of the reaction, without implying any assumption on the nature of the microscopic rates contributing to the K_D_ value itself.

Variation of the ATPase reaction as a function of time was fitted to the mixed-exponential equation:
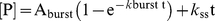
(3)where [P] is the concentration of products (pmols) at any given time point, *t* is time, A_burst_ is the burst amplitude, *k*_burst_ is the rate of the exponential phase, *k*_ss_ is the rate of the steady-state linear phase. The burst amplitude reflects the amount of enzyme-substrate complex reacting in the exponential phase, hence it has the dimension of a concentration. The burst rate is the combined rate of product formation (Pi) and release during the exponential phase, hence the dimensions of a rate constant (s^−1^).

Variation of the amount of unwound RNA substrate as a function of time was fitted to a simple exponential equation:
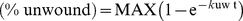
(4)where MAX is the the amount of unwound substrate extrapolated at infinite time, *k_uw_* is the apparent rate of unwinding.

The eq. dissociation constant for the binding of DDX3 to its substrate ATP or RNA in the absence of catalysis was anlayzed as a function of variable enzyme concentrations according to a simple binding equilibrium:
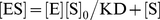
(5)where [ES] is the enzyme-substrate formed at equilibrium, [S]_0_ is the input substrate and [E] is the variable enzyme concentration. According to the Briggs-Haldane equilibrium (Eq. 1), it holds the following relationship:
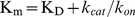
(6)where *k_on_* is the apparent rate of substrate binding. Thus, K_m_>K_D_.

### RNA pull down assay

Talon beads (Dynal) were incubated over night at 4°C with 1 µg of purified DDX3wt or DDX3ΔINS proteins, respectively. After washing three times, 1 mg of HIV-1Lai genomic RNA, prepared from HIV-1 virions with the QiaAmp viral RNA Mini kit (Qiagen), was added for 2 hours. After 3 washing steps 100 µl H_2_O was added and bound RNA was extracted with the RNAeasy kit (Qiagen). The extracted RNA was analyzed by RT-PCR using random hexamer primers for RT and *gag*-specific primers for PCR (HIV-1 *gag* fw: GAGGAAGCTGCAGAATGGG; HIV-1 *gag* rev: GGTCCTTGTCTTATGTCCAGAAT-GCTG). PCR conditions were 3 min 95°C followed by 40 cycles of 30 sec 95°C, 30 sec 50°C, 30 sec 72°C, followed by 7 min 72°C. An aliquot of 50% of the input RNA was kept as RT-PCR control and viral HIVC-1 Lai plasmid was used as positive control for PCR. The experiment was performed three times independently.

### Selection of peptides by phage display

Three different random peptide phage libraries (PhD-7, PhD-C7, PhD-12, New England Biolabs) were used. For positive selections we used the DDX3 insert peptide (GSGSGS-*HHHHHH*-GSGSGS-**ALRAMKENGR**-GSGSGS-*EQKLISEEDL*), for negative selections a scrambled version of the DDX3 insert (GSGSGS-*HHHHHH*-GSGSGS-**RKAEGLNARM**-GSGSGS-*EQKLISEEDL*), each with a His-tag for immobilization and a Myc-tag for detection (tags are in italics, the actual DDX3 peptide sequences in bold). Peptides (1 µg) were coated on TALON beads over night at 4°C. After 6 washes (PBS/0.25% gelatine/0.1%Tween20), 10 µl of each of the original phage libraries were added to the beads and rotated over night at 4°C. After extensive washing, bound phages were eluted by pH shift (0.2 M glycine/HCl, pH 2.2), neutralized with 1 M Tris-HCl (pH 9.1) and used for negative selection as described above. Phage in supernatants after negative selection were amplified in E. coli ER2738, precipitated over night and used for two additional selection rounds. Finally, phages were titered, single clones were picked, amplified and DNA was prepared for sequence analysis.

### Peptide inhibition experiments

HelaP4 cells were seeded at 1×10^4^ cells/well in DMEM (10%FCS, 2% L-glutamine, 1% penicillin/streptamycine) and infected with HIV-1 Lai (MOI of 0.3) over night at 37°C. 24 hours after infection, cells were washed with PBS and different amounts (5, 20, 50 µM in DMEM) of peptides (DDX3-INS1: SDVPTQV-GGRRRRRRRRR and control: GSGSGS-GGRRRRRRRRR) were added in fresh medium without FCS for 4 hours. Subsequently medium was again supplemented with 10% FCS and cells were incubated for further 24 hours at 37°C. The next day, viral supernatants were collected and titered on Tzm-bl cells in the presence of polybrene (16 µg/ml). 44 hours after infection, supernatants were discarded, cells were washed with PBS, lysed for 10 min at room temperature in harvest buffer (50 mM MES/Tris pH 7.8/10% glycerol/0.1% triton X100/1 µM DTT) and frozen at −80°C. Luciferase activity in the lysates was measured by luminometry (Lumistar Galaxy, BMG Labbiotechnologies Germany).

## References

[1] Cordin O, Banroques J, Tanner NK, Linder P (2006). The DEAD-box protein family of RNA helicases.. Gene.

[2] Abdelhaleem M, Maltais L, Wain H (2003). The human DDX and DHX gene families of putative RNA helicases.. Genomics.

[3] Fuller-Pace FV (2006). DExD/H box RNA helicases: multifunctional proteins with important roles in transcriptional regulation.. Nucleic Acids Res.

[4] Linder P (2006). Dead-box proteins: a family affair–active and passive players in RNP-remodeling.. Nucleic Acids Res.

[5] Schütz P, Karlberg T, van den Berg S, Collins R, Lehtiö L (2010). Comparative structural analysis of human DEAD-box RNA helicases.. PLoS One.

[6] Sengoku T, Nureki O, Nakamura A, Kobayashi S, Yokoyama S (2006). Structural basis for RNA unwinding by the DEAD-box protein Drosophila Vasa.. Cell.

[7] von Moeller H, Basquin C, Conti E (2009). The mRNA export protein DBP5 binds RNA and the cytoplasmic nucleoporin NUP214 in a mutually exclusive manner.. Nat Struct Mol Biol.

[8] Ariumi Y, Kuroki M, Abe K, Dansako H, Ikeda M (2007). DDX3 DEAD-box RNA helicase is required for hepatitis C virus RNA replication.. J Virol.

[9] Kalverda AP, Thompson GS, Vogel A, Schroder M, Bowie AG (2009). Poxvirus K7 protein adopts a Bcl-2 fold: biochemical mapping of its interactions with human DEAD box RNA helicase DDX3.. J Mol Biol.

[10] Kwong AD, Rao BG, Jeang KT (2005). Viral and cellular RNA helicases as antiviral targets.. Nat Rev Drug Discov.

[11] Wang H, Kim S, Ryu WS (2009). DDX3 DEAD-Box RNA helicase inhibits hepatitis B virus reverse transcription by incorporation into nucleocapsids.. J Virol.

[12] Maga G, Falchi F, Garbelli A, Belfiore A, Witvrouw M (2008). Pharmacophore modeling and molecular docking led to the discovery of inhibitors of human immunodeficiency virus-1 replication targeting the human cellular aspartic acid-glutamic acid-alanine-aspartic acid box polypeptide 3.. J Med Chem.

[13] Yedavalli VS, Neuveut C, Chi YH, Kleiman L, Jeang KT (2004). Requirement of DDX3 DEAD box RNA helicase for HIV-1 Rev-RRE export function.. Cell.

[14] König R, Zhou Y, Elleder D, Diamond TL, Bonamy GM (2008). Global analysis of host-pathogen interactions that regulate early-stage HIV-1 replication.. Cell.

[15] Franca R, Belfiore A, Spadari S, Maga G (2007). Human DEAD-box ATPase DDX3 shows a relaxed nucleoside substrate specificity.. Proteins.

[16] Yedavalli VS, Zhang N, Cai H, Zhang P, Starost MF (2008). Ring expanded nucleoside analogues inhibit RNA helicase and intracellular human immunodeficiency virus type 1 replication.. J Med Chem.

[17] Tarn WY, Chang TH (2009). The current understanding of Ded1p/DDX3 homologs from yeast to human.. RNA Biol.

[18] Hogbom M, Collins R, van den Berg S, Jenvert RM, Karlberg T (2007). Crystal structure of conserved domains 1 and 2 of the human DEAD-box helicase DDX3X in complex with the mononucleotide AMP.. J Mol Biol.

[19] Kikuma T, Ohtsu M, Utsugi T, Koga S, Okuhara K (2004). Dbp9p, a member of the DEAD box protein family, exhibits DNA helicase activity.. J Biol Chem.

[20] Chen Y, Potratz JP, Tijerina P, Del Campo M, Lambowitz AM (2008). DEAD-box proteins can completely separate an RNA duplex using a single ATP.. Proc Natl Acad Sci U S A.

[21] Hilbert M, Karow AR, Klostermeier D (2009). The mechanism of ATP-dependent RNA unwinding by DEAD box proteins.. Biol Chem.

[22] Liu F, Putnam A, Jankowsky E (2008). ATP hydrolysis is required for DEAD-box protein recycling but not for duplex unwinding.. Proc Natl Acad Sci U S A.

[23] Yang Q, Del Campo M, Lambowitz AM, Jankowsky E (2007). DEAD-box proteins unwind duplexes by local strand separation.. Mol Cell.

[24] Liu J, Henao-Mejia J, Liu H, Zhao Y, He JJ (2011). Translational Regulation of HIV-1 Replication by HIV-1 Rev Cellular Cofactors Sam68, eIF5A, hRIP, and DDX3.. J Neuroimmune Pharmacol.

