# Correction: A motif unique to the human dead-box protein DDX3 is important for nucleic acid binding, ATP hydrolysis, RNA/DNA unwinding and HIV-1 replication

**DOI:** 10.1371/journal.pone.0341385

**Published:** 2026-01-26

**Authors:** The PLOS One Editors

After publication of this article [1], concerns were raised about Figs 2–4.

**Fig 2:**
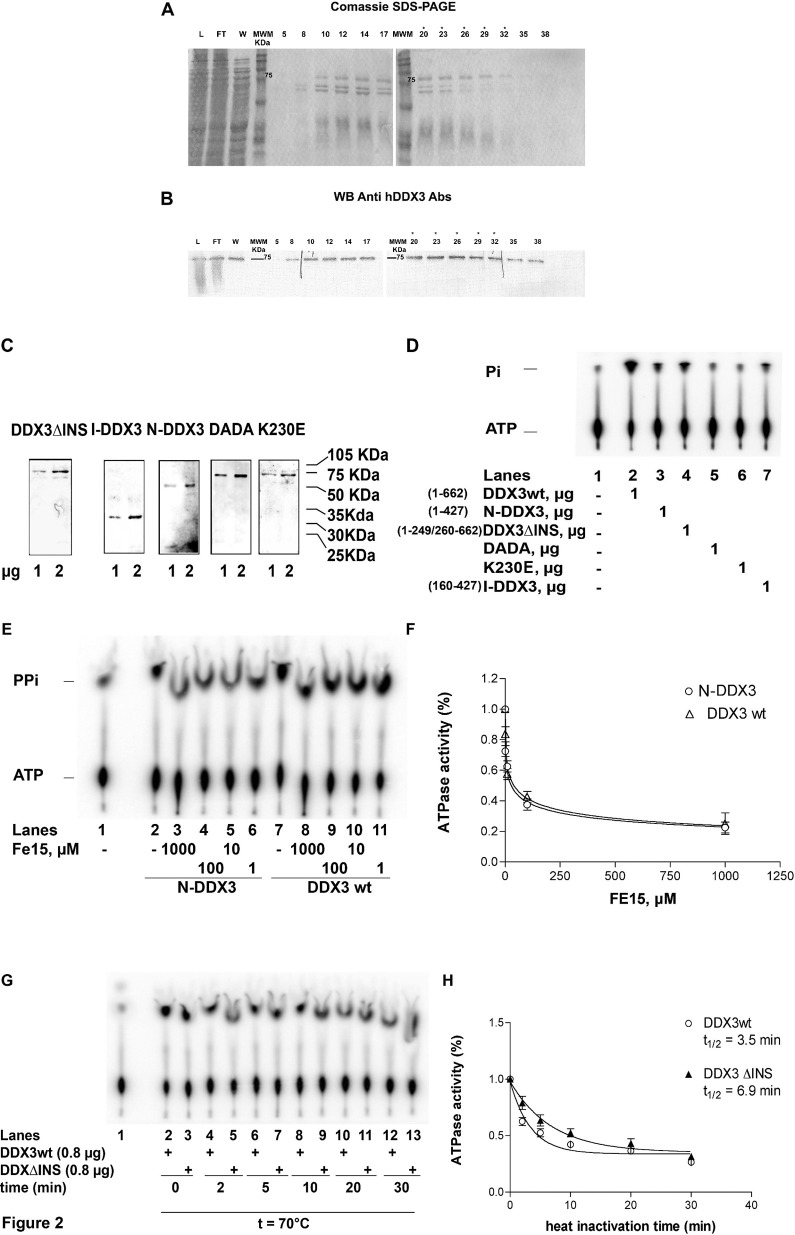
Purification of recombinant human DDX3. **A.** Coomassie staining of SDS-PAGE of the full length human DDX3 containing fractions eluted from the hydroxyhapatite column. L, loading; FT, flow-through; W, wash. Molecular weight is indicated alongside. Fraction numbers are on top. Asterisks indicate the fractions used for the experiments. **B.** Western blot analysis of the fractions shown in panel A with anti-human DDX3 polyclonal antibodies. **C.** Coomassie staining of SDS-PAGE of the final purified preparation of all the recombinant DDX3 proteins. **D.** ATPase activity of all the recombinant DDX3 proteins. Reactions were performed as described in Material and Methods in the absence of nucleic acids. Unreacted substrate (ATP) was separated from the product (phosphate, Pi) by thin layer chromatography. The length (amino acids) of each protein is indicated in brackets. **E.** ATPase activity of N-DDX3 (lanes 2–6) or DDX3wt (lanes 7–11) in the absence (lanes 2 and 7) or in the presence of increasing amounts of the DDX3 inhibitor FE15. Lane 1, control without enzyme. **F.** Dose-response curves for the inhibition by FE15 of the ATPase activity of DDX3wt (circles) or N-DDX3 (triangles). Values are means of three independent determinations. Error bars are ± S.D. **G.** Representative time course experiment of the ATPase activity of DDX3wt (even lanes) or DDXΔINS (odd lanes) at 70°C. Lane 1, control without enzyme. **H.** Activity decay curves for the ATP hydrolysis by DDX3wt (circles) or DDX3ΔINS (triangles). Curves were fitted to a simple exponential decay model. Values are means of three independent determinations. Error bars are ± S.D.

**Fig 3:**
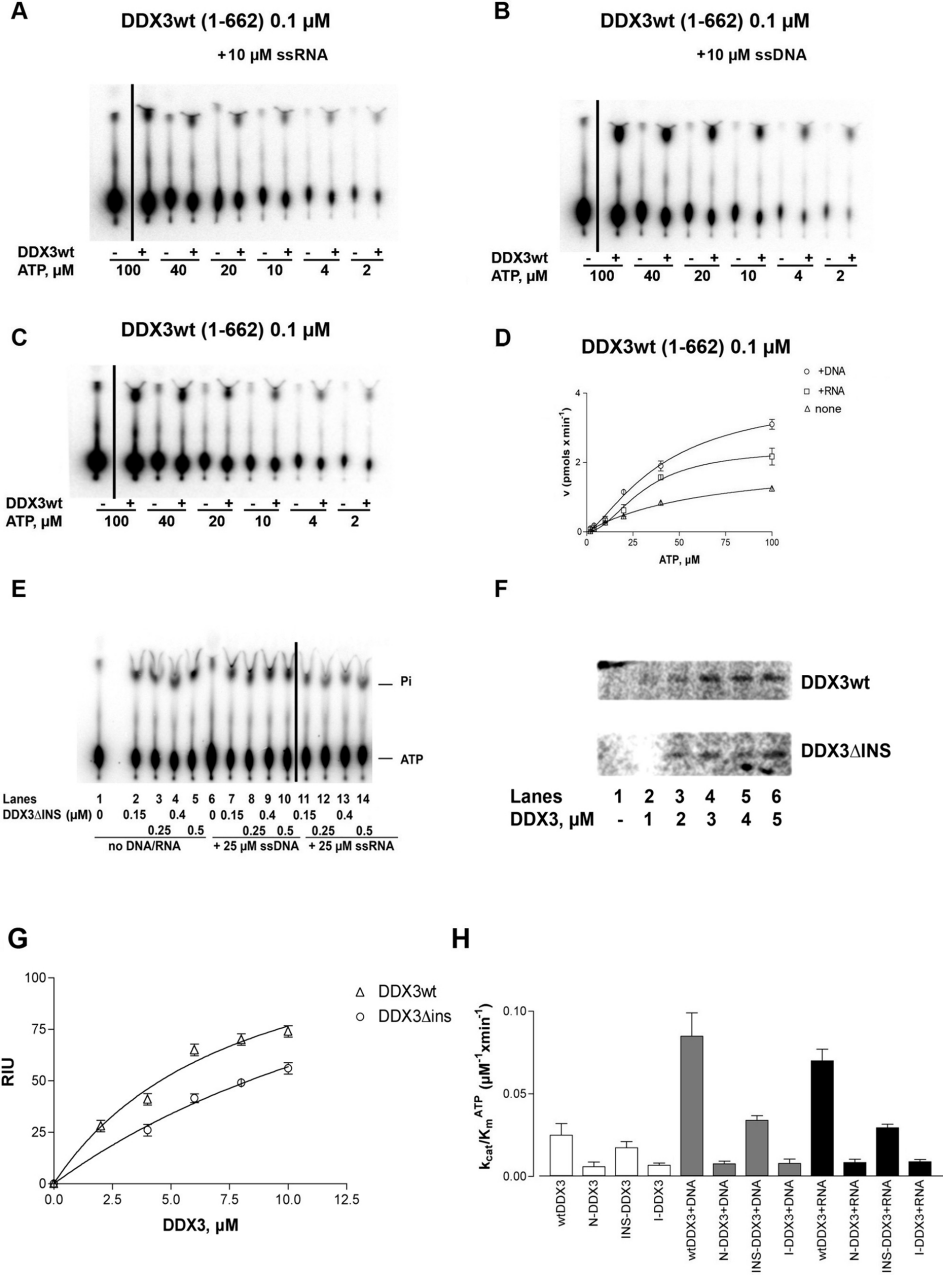
Characterization of the ATPase activity of recombinant human DDX3. Reactions were performed as described in Material and Methods. **A.** Product analysis of a representative experiment for the ATPase reaction catalyzed by 0.1 µM full length DDX3 in the presence of 10 µM ss RNA. **B.** As in panel A, but in the presence of 10 µM ss DNA. **C.** As in panel A, but in the absence of nucleic acids. Black lines in each experiment indicate the location of lanes spliced together from the same plate. **D.** Variation of the initial velocities of the reaction as a function of ATP concentrations, in the absence (triangles) or in the presence of 10 µM ss DNA (circles) or 10 µM ss RNA (squares). Data were fitted to Eq.(1) (see Materials and Methods). Values are the means of three independent determinations. Error bars are ± SD. **E.** Product analysis of the ATPase reaction catalyzed by increasing amounts of the DDX3ΔINS mutant in the absence (lanes 2–5) or in the presence of 25 µM ss DNA (lanes 7–10) or ssRNA (lanes 11–14). Lanes 1 and 6, control reactions in the absence of enzyme. Black lines indicate the location of lanes spliced together from the same plate. **F.** [α-^33^P] ATP was UV crosslinked to increasing amounts of DDX3wt (upper panel) or ΔINS mutant (lower panel). Radioactive proteins were resolved on SDS-PAGE and revealed by phosphoImaging. **G.** Binding of DDX3 to ATP, as revealed by UV-crosslinking. Data were fitted to Eq.(5) (see Materials and Methods). Values are the means of three independent determinations. Error bars are ± SD. **H.** Comparison of the catalytic efficiencies (k_cat_/K_m_) for ATP hydrolysis of DDX3wt (aa 1–662), and the N-DDX3 (aa 1–427), DDX3ΔINS and I-DDX3 (aa 160–427) mutants, in the absence (white bars) or in the presence of ssDNA (grey bars) or ssRNA (black bars). Determination of the kinetic constants k_cat_ and K_m_ was performed as described in Materials and Methods. Values represent the means between two independent estimates of the k_cat_/K_m_ values from two sets of experiments. Error bars represent ±SD.

**Fig 4:**
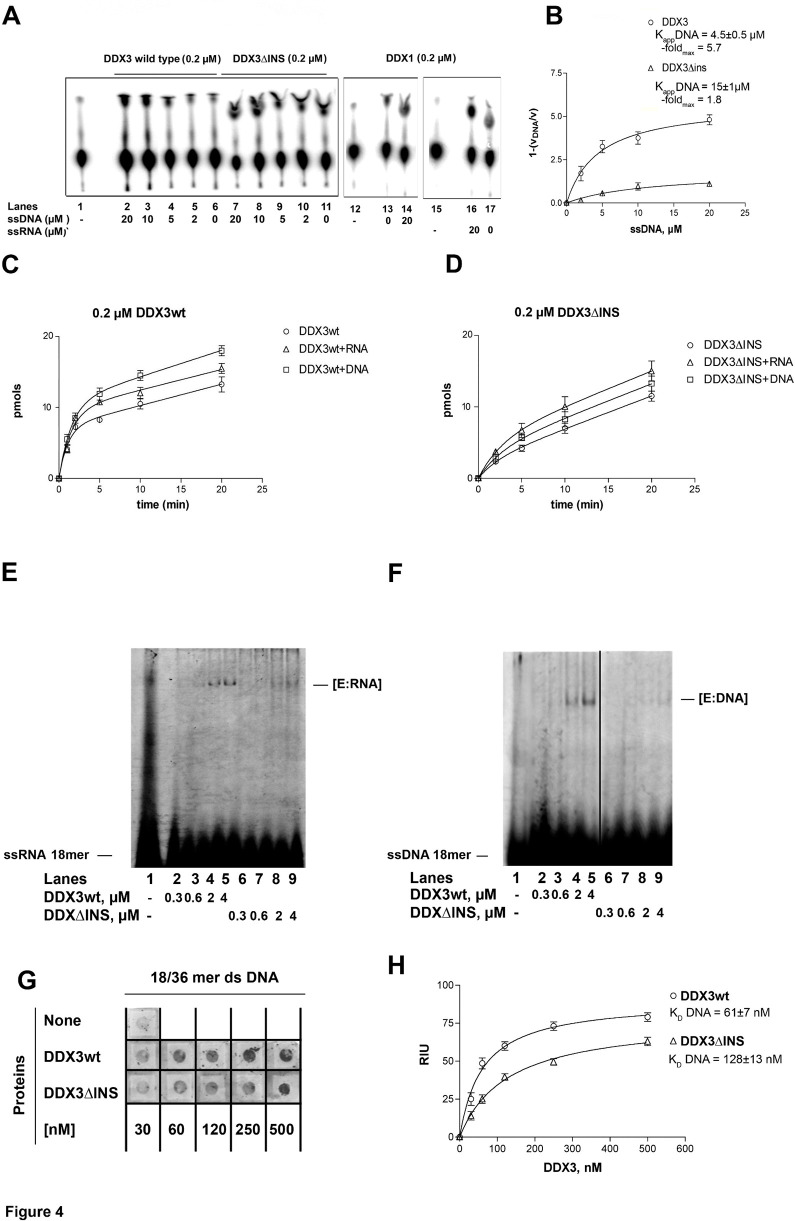
The specific insertion of human DDX3 is important for nucleic-acid stimulation of ATPase activity. Reactions were performed as described in Material and Methods. **A.** Product analysis for the ATPase reaction catalyzed by the DDX3wt (lanes 2–6) and the DDX3ΔINS (lanes 7–11) mutant proteins in the absence (lanes 6, 11) or in the presence of increasing concentrations of ssDNA. Reactions with the full length DDX1 protein (lanes 12–17) in the absence (lane 13, 17) or in the presence of a fixed amount of DNA (lane 14) or RNA (lane 16), were included for comparison. Lanes 1, 12 and 15, control reactions without enzymes. Assays with the full length DDX1 protein derived are from two additional assays, such that Fig 4A is composed of images from 3 assays. **B.** Variation of the increase in the ATPase reaction rate (Δv) as a function of the ssDNA concentration in the presence of DDX3wt (circles) or DDX3ΔINS (triangles). The Δv values were derived as described in Materials and Methods. Data were fitted to Eq.(2) (see Materials and Methods). Values are the means of three independent determinations. Error bars are ± SD. **C.** Progress curves for the product formation during the ATPase reaction catalyzed by 0.2 µM of DDX3wt as a function of time, in the absence (circles) or in the presence of 10 µM ssRNA (triangles) or 10 µM ssDNA (squares). Data were fitted to Eq. (3) (see Materials and Methods). Values are the means of three independent determinations. Error bars are ± SD. **D.** As in panel C, but in the presence of 0.2 µM of the DDX3ΔINS mutant. **E.** Increasing amounts of DDX3wt (second row) or DDX3ΔINS mutant (third row), were incubated with a fixed concentration of (6-FAM)-5′-labelled ssDNA oligonucleotide. Nitrocellulose filter bound protein-DNA complexes were revealed by laser scanning. First row, control in the absence of proteins. **F.** Binding of DDX3 to ssDNA, as revealed by filter-binding assays. Data were fitted to Eq.(5) (see Materials and Methods). Values are the means of three independent determinations. Error bars are ± SD. The black line indicates the location of lanes spliced together following rearrangement of lanes from the same assay. **G.** Increasing amounts of DDX3wt (lanes 2–5) or DDX3ΔINS (lanes 6–9) were incubated in the presence of a (6-FAM)-5′-labelled ss RNA oligonucleotide. Enzyme-RNA ([E:RNA]) complexes were resolved by non denaturing PAGE and visualized by laser scanning. Lane 1, oligonucleotide alone.

Specifically, when color levels and/or aspect ratio are altered:

- Fig 2A in [1] appears similar to Fig 1B in [2], despite representing different experimental conditions.
- In Figs 2D and 3A–3B there are vertical discontinuities between lanes 1 and 2.
- There appear to be vertical discontinuities in the following panels:
  - In Fig 3C, between lanes 1 and 2.
  - In Fig 3E, between lanes 10 and 11.
  - In Fig 4A, between lanes 11–12 and 13–14.
- There are two errors in the lane numbers of the caption for Fig 3E. Please see the complete, correct Fig 3 caption below.

In editorial follow-up, regarding Fig 2A, the first author AG stated that the incorrect image was erroneously included in [1] when preparing the originally published figure, and provided the original image panels intended to be included in Fig 2A of [1]. They stated that the fractions used to conduct subsequent experiments (marked with an asterisk) were chosen based on the quantity and quality of protein purified from each fraction, and as such the replicate data for this figure provided in S1 File show different fractions with strongest signal. They also stated that the vertical discontinuities in Figs 3A–3C were due to the removal of a blank lane between lanes 1 and 2, as supported by the underlying images; they indicated this may also apply to Fig 2D. Regarding the vertical discontinuity in Fig 3E, the first author stated this was likely due to removal of a repeated control lane.

The first author AG stated that Fig 4A was composed of images originating from three separate thin layer chromatography (TLC) plates. The explanations provided are supported by the available original and repeat image data underlying these figures. The original images underlying Figs 2D and 3E are not available; however, PLOS considers the explanation consistent with the information and data provided for the other figure panels.

Additionally, the first author AG notified the journal that lanes 2–5 in Fig 4F have been swapped with lanes 6–9 so that the lane order was consistent with Fig 4E. With this Correction, the first author AG has provided updated Figs 2–4 and associated figure captions, with splicing between lanes from the same scanned TLC plates clearly marked and lanes derived from separate TLC plates presented in separate panels, each with the control lane from the same plate.

The first author AG stated that the original image data are unavailable for Figs 2D, 3E, 4B–4D, 4G–4H, 6A–6B, and 7, and no quantitative data are available for Figs 2–5 and 7. They stated that original image data are available for Figs 2E, 2G, 3A–3C, 3F, 4A, 4E–4F, and 5A–5B, that both original and repeat data are available for Figs 2A–2B and repeat data only are available for Fig 2C and 3E, stated to be from experiments completed during the same period as the original experiments.

The authors apologize for the errors in the published article.

The corresponding author is deceased. Correspondence regarding this article may be sent to the first author, AG (anna.garbelli@cnr.it).

## Supporting information

S1 File Underlying and replicate data. This file includes the original figure prepared for Fig 2, the original underlying image data for Figs 2A–B (photographs of laboratory notebook), 2E, 2G, 3A–C, 3F, 4A, 4E–F and 5A–B. Repeat data underlying Figs 2A–C, and 3E.
(PDF)
