# Supplementary material for: Correction: A motif unique to the human dead-box protein DDX3 is important for nucleic acid binding, ATP hydrolysis, RNA/DNA unwinding and HIV-1 replication
Source: PLoS One. 2026 Jan 26;21(1):e0341385. doi: 10.1371/journal.pone.0341385 (PMC12834386; doi:10.1371/journal.pone.0341385)
Supplement: S1 File — This file includes the original figure prepared for Fig 2, the original underlying image data for Figs 2A–B (photographs of laboratory notebook), 2E, 2G, 3A–C, 3F, 4A, 4E–F and 5A–B. Repeat data underlying Figs 2A–C, and 3E. (PDF) [file pone.0341385.s001.pdf]

Supporting information: A Motif Unique to the Human Dead-Box Protein DDX3 Is Important for Nucleic Acid Binding, ATP Hydrolysis, RNA/DNA Unwinding and HIV-1 Replication

Fig.2

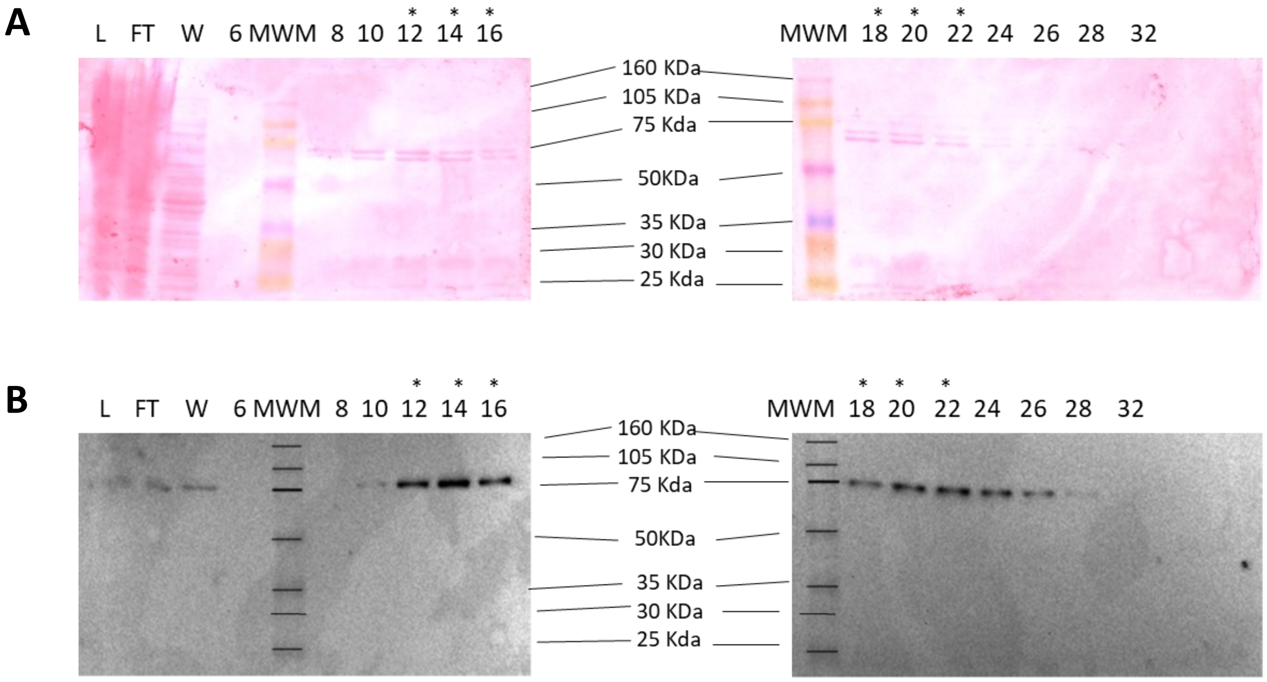

Figure2A-B. Purification of recombinant human DDX3. These are alternative data obtained from a replicated experiment, performed using the same protocol as the one presented into the main text. **A.** Ponceau staining of SDS-PAGE of the full length human DDX3 containing fractions eluted from the hydroxyhapatite column. L, loading; FT, flow-through; W, wash; MWM, Molecular weight marker. **B.** Western blot analysis of the fractions shown in panel A with anti-human DDX3 polyclonal antibody. Asterisks indicated the fractions used for the experiments.

E

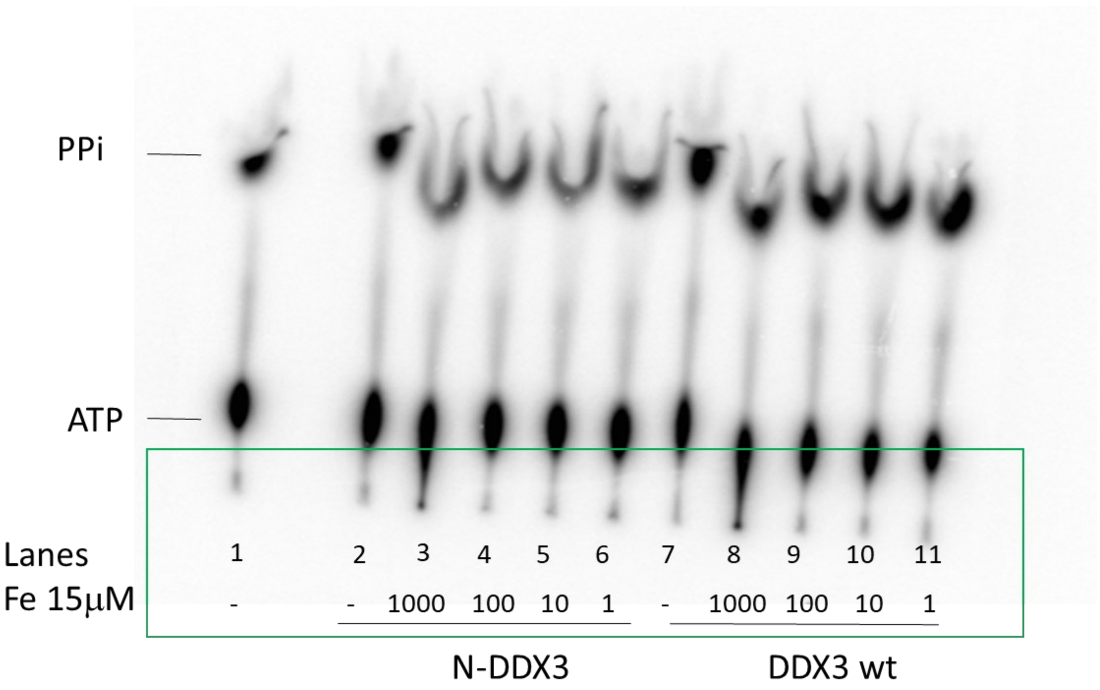

Fig. 2E. Uncropped scan (in green box) of the assay presented in the paper.

G

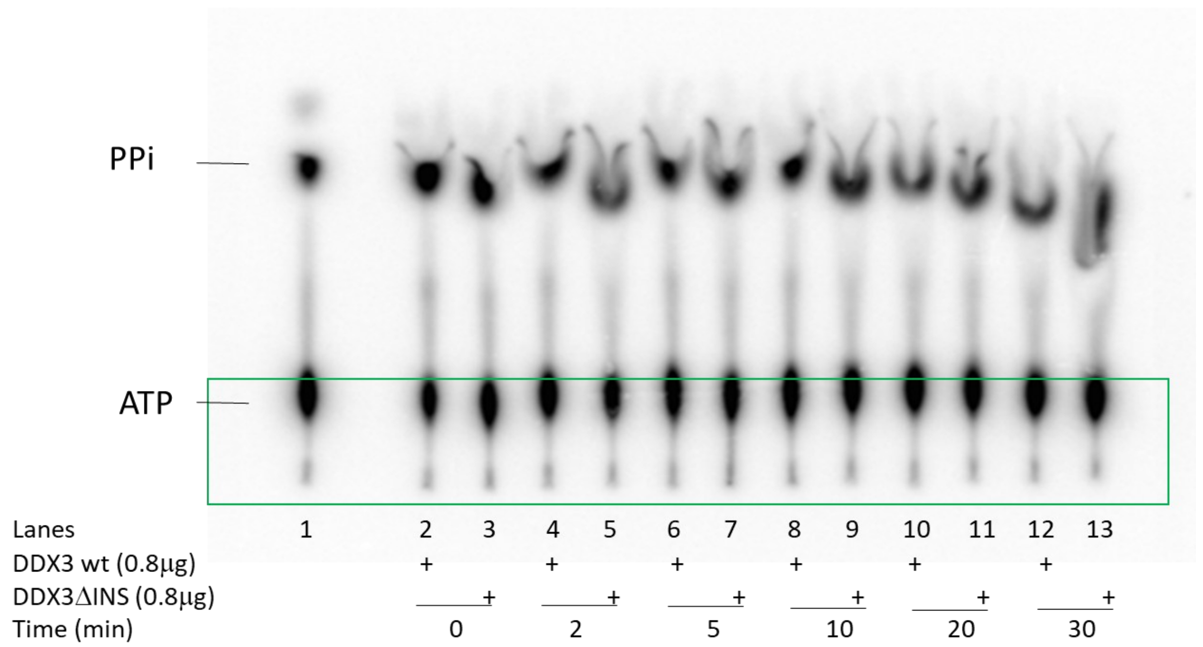

Fig. 2G. Uncropped scan (in green box) of the assay presented in the paper. In green box is possible appreciated that horizontal discontinuity at the bottom of the panel is a common feature of this kind of assays that migrate by capillarity. Discontinuity could depend from the amount of buffer added for migration. It is possible observe the same detail also in other assays presented in this document (see fig. 2E, 3A, 3B, 3C). In the paper maybe this discontinuity is more accentuated due to the contrast used.

Fig.3

A **DDX3 wt (1-662) 0.1μM**

+ 10 μM ssRNA

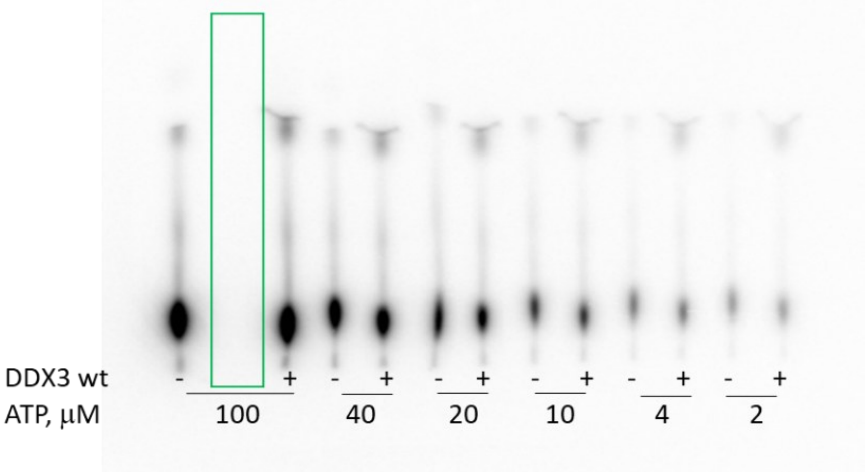

B **DDX3 wt (1-662) 0.1μM**

+ 10 μM ssDNA

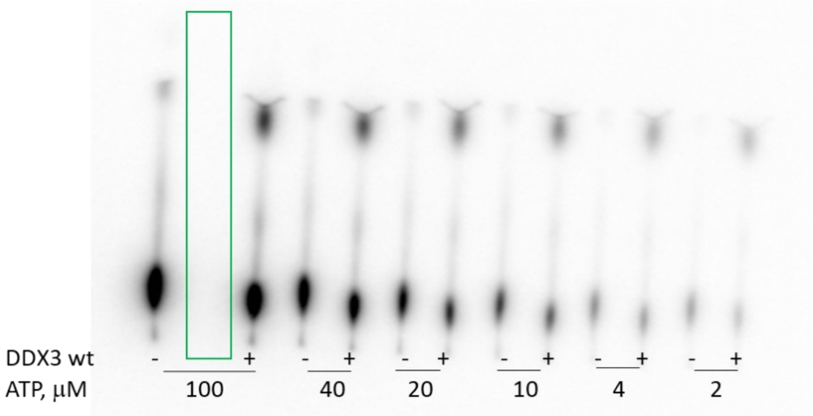

CNR – Istituto di Genetica Molecolare
Luigi Luca Cavalli-Sforza
Via Abbiategrasso, 207 - 27100 Pavia, Italia
Tel: 0382 546322– Fax: 0382 546370

C **DDX3 wt (1-662) 0.1μM**

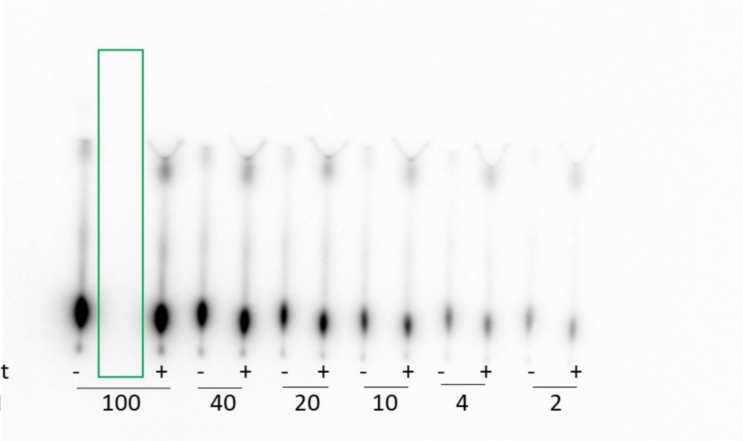

Fig. 3A-C. Original uncropped scan (in green box) and less contrasted version of the assays presented in the paper. In green box you can appreciated that vertical discontinuities between lane 1 and 2 of all these assays is due to the cropping of an empty area.

E

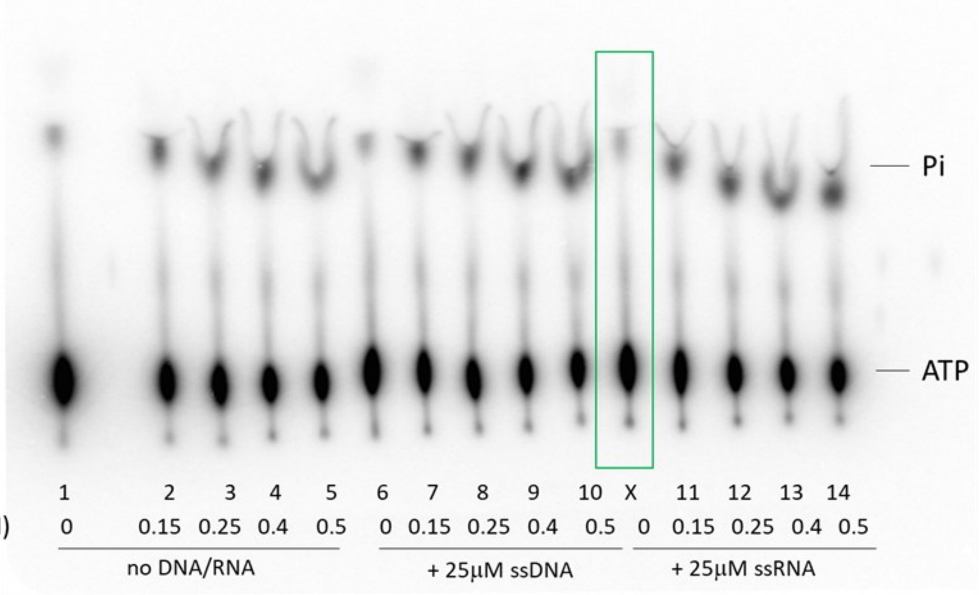

Fig. 3E. Original uncropped scan and less contrasted version of a similar assays that we present in the paper. This image is a replicate of the one presented and probably, as in this case, also for the assay of Fig. 3E of the paper, the vertical discontinuity is likely due to removal of a repeated control lane (lane X in the green box).

F

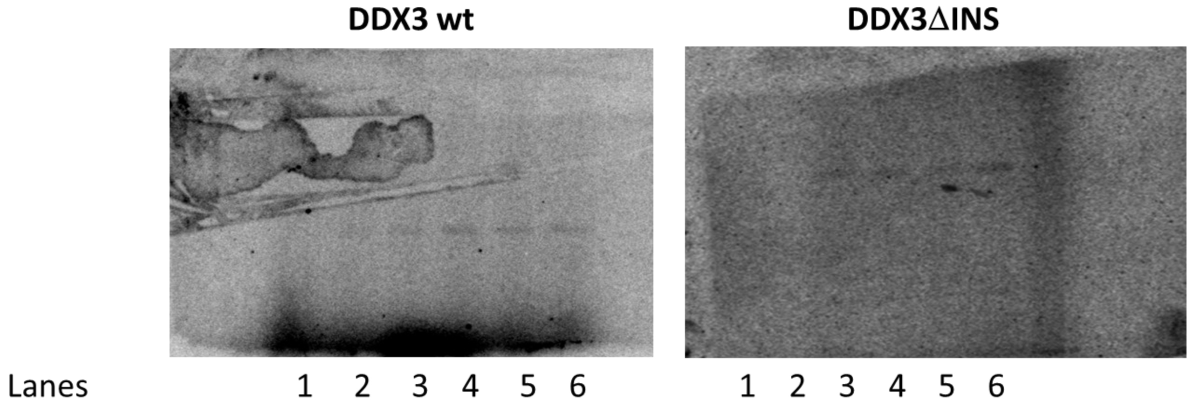

Fig. 3F: Original uncropped and less contrasted scan of the same assays presented in the paper. We had to contrast the images a bit to allow the bands to be seen.

Fig.4

a) samples 1-11

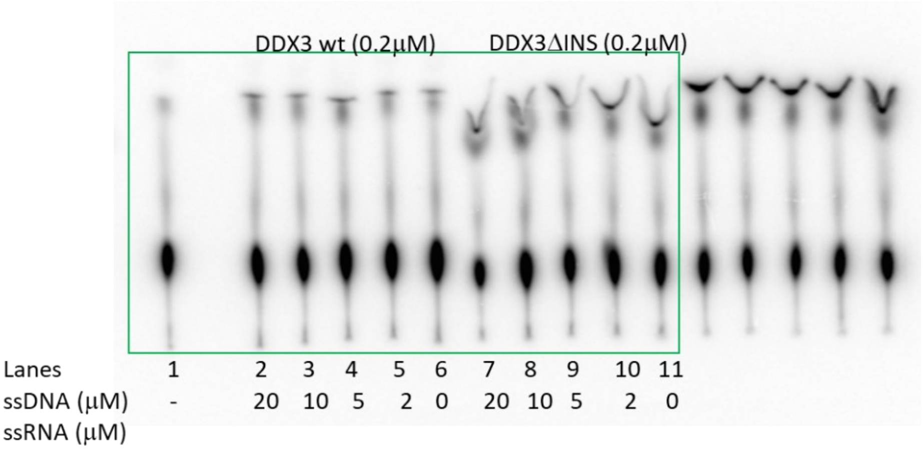

b) samples 12-13-14

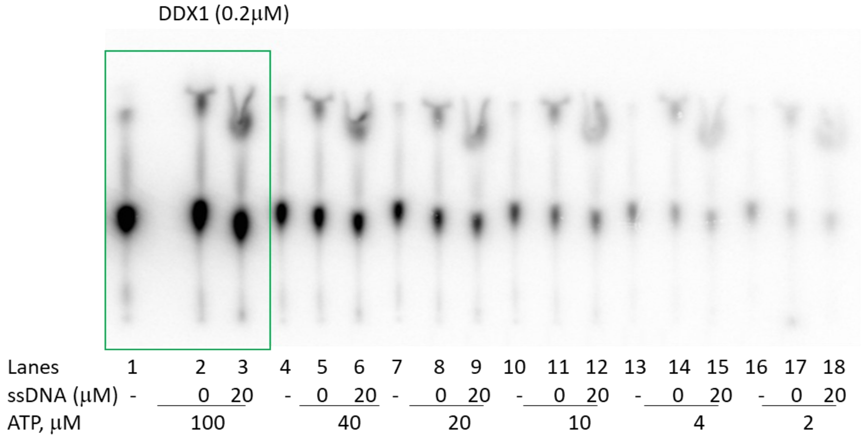

c) samples 15-16-17

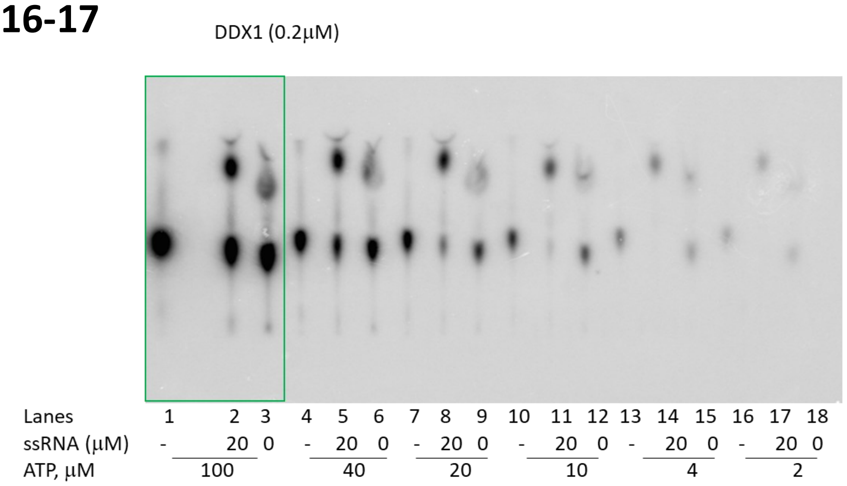

Fig. 4A: Uncropped scan and less contrasted version of the same assays presented in the paper. In green boxes you can appreciated the samples shown in the Figure 4A of the paper. In green boxes: a) samples 1-11; b) samples 12-14, c) samples 15-17.

E

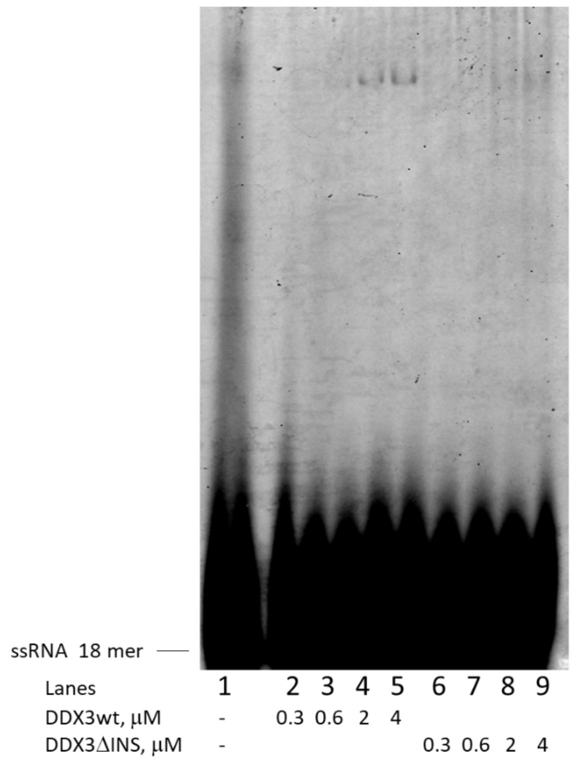

F

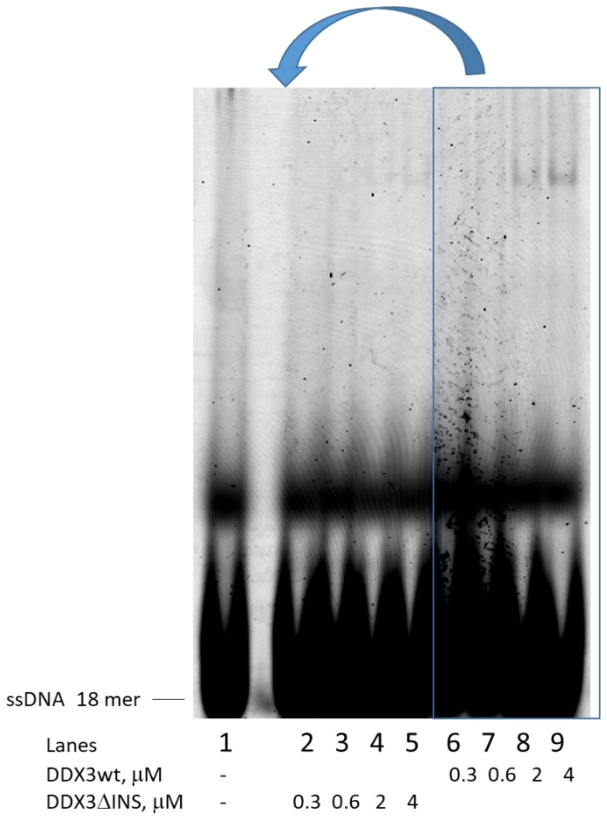

Fig. 4E: Uncropped scan and less contrasted version of the same assays presented in the Figure 4E and F of the paper. The image presented in 4F has been modified for aesthetic purposes so that both assays can be read in the same order.

CNR – Istituto di Genetica Molecolare
Luigi Luca Cavalli-Sforza
Via Abbiategrasso, 207 - 27100 Pavia, Italia
Tel: 0382 546322– Fax: 0382 546370

Fig.5

A

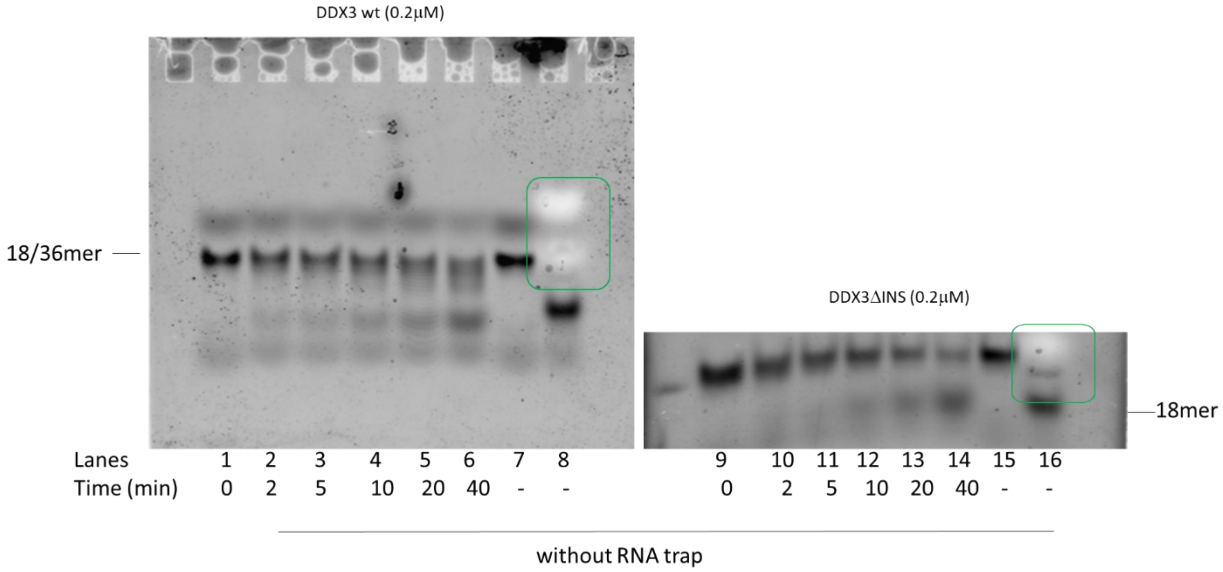

B

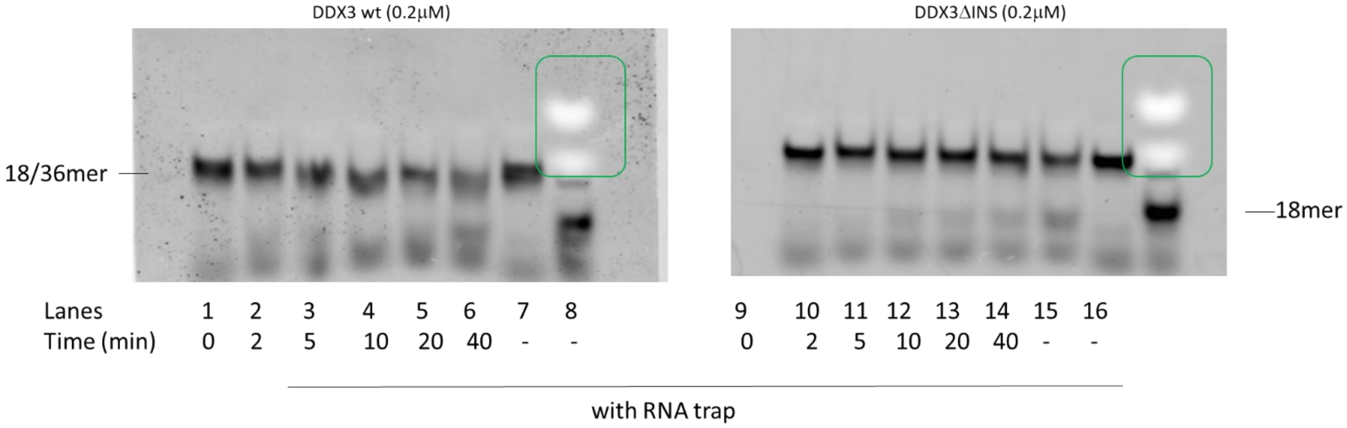

Fig. 5: A. Uncropped scan and less contrasted version of DDX3wt gel, cropped scan and less contrasted version of DDX3ΔINS gel presented in the Figure 5A of the paper. **B.** Uncropped scan and less contrasted version of both assays presented in the Figure 5B of the paper. In green boxes are pointed out the common feature for boiled samples that usually presented white stained in the upper part.

Fig. 2

A

A

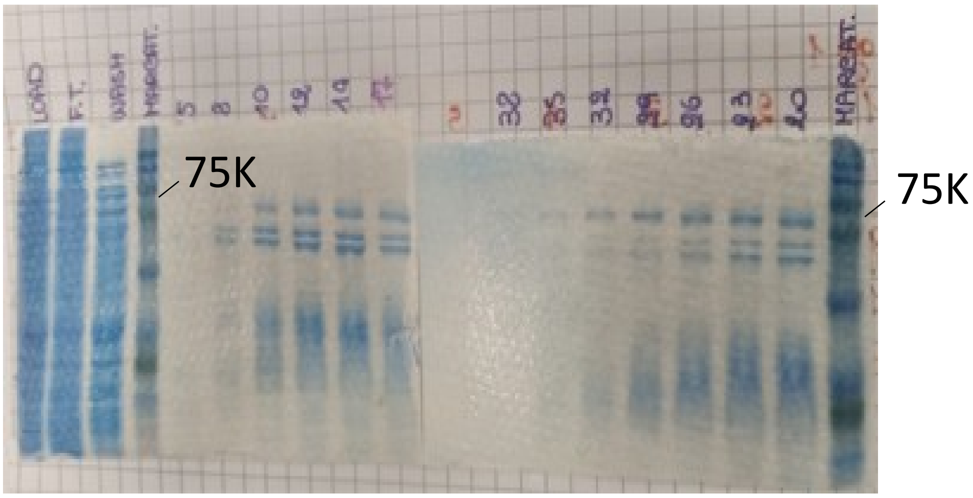

B

B

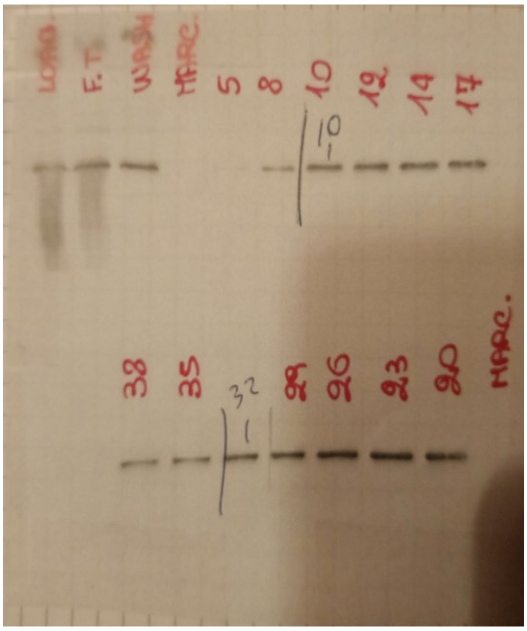

Figure2A-B: **A.** Coomassie staining and **B.** Western blot of the full length human DDX3. These images represent the experiment presented in Figures 2A and B, which we originally intended to present in the main text, and which can be found in this revised version. The images were photographed from the lab book.

C **DDX3ΔINS**

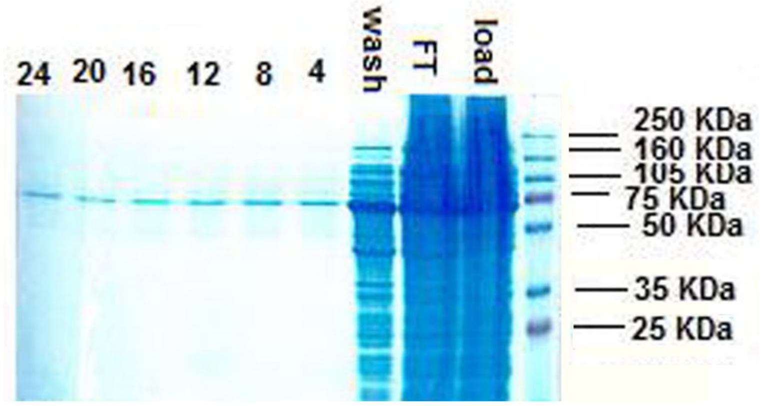

I-DDX3

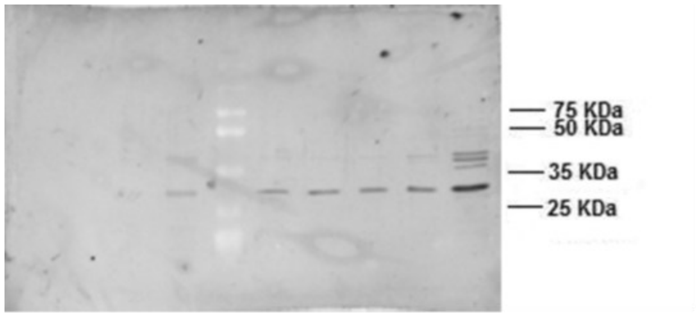

N-DDX3

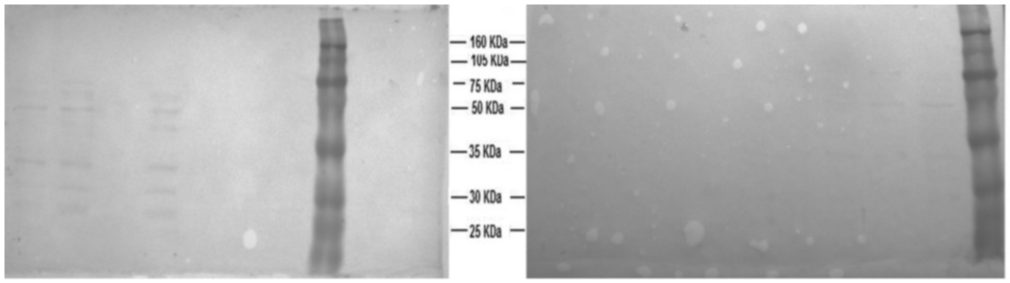

DADA

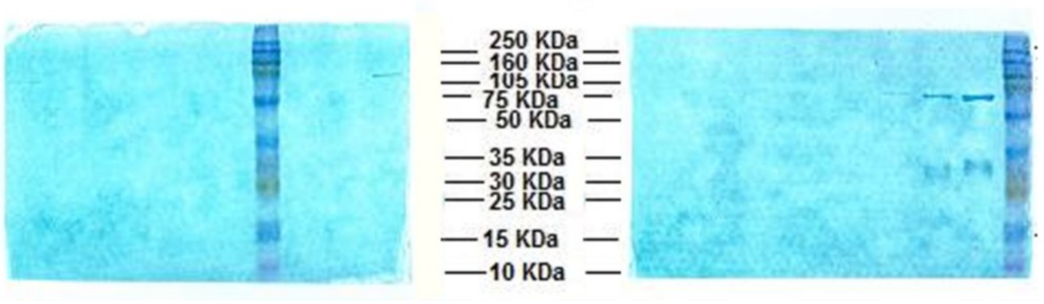

K230E

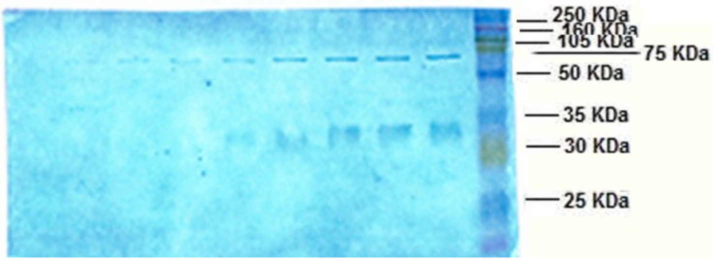

Fig. 2C. Coomassie and Ponceau staining. of the purified preparation of the recombinant DDX3 proteins. Only the Western blot analysis image is available for the I-DDX3 sample.
